# Specific genomic and transcriptomic aberrations in tumors induced by partial hepatectomy of a chronically inflamed murine liver

**DOI:** 10.18632/oncotarget.2515

**Published:** 2014-09-25

**Authors:** Ezra Ella, Denise Heim, Evgeniy Stoyanov, Rona Harari-Steinfeld, Israel Steinfeld, Orit Pappo, Temima Schnitzer Perlman, Natalie Nachmansson, Ludmila Rivkin, Devorah Olam, Rinat Abramovitch, Henning Wege, Eithan Galun, Daniel Goldenberg

**Affiliations:** ^1^ The Goldyne Savad Institute of Gene Therapy, Hadassah-Hebrew University Medical Center, Jerusalem, Israel; ^2^ Department of Gastroenterology and Hepatology, University Medical Center Hamburg-Eppendorf, Hamburg, Germany; ^3^ Computer Science Department, Technion-Israel Institute of Technology, Haifa, Israel; ^4^ Department of Pathology, Hadassah-Hebrew University Medical Center, Jerusalem, Israel; ^5^ Magnetic Resonance Imaging/Magnetic Resonance Spectroscopy Laboratory, Human Biology Research Center, Hadassah-Hebrew University Medical Center, Jerusalem, Israel

**Keywords:** HCC, liver regeneration, chronic hepatitis, genomic instability, Crem

## Abstract

Resection of hepatocellular carcinoma (HCC) tumors by partial hepatectomy (PHx) is associated with promoting hepatocarcinogenesis. We have previously reported that PHx promotes hepatocarcinogenesis in the Mdr2-knockout (Mdr2-KO) mouse, a model for inflammation-mediated HCC. Now, to explore the molecular mechanisms underlying the tumor-promoting effect of PHx, we compared genomic and transcriptomic profiles of HCC tumors developing in the Mdr2-KO mice either spontaneously or following PHx. PHx accelerated HCC development in these mice by four months. PHx-induced tumors had major chromosomal aberrations: all were amplifications affecting multiple chromosomes. Most of these amplifications were located near the acrocentric centromeres of murine chromosomes. Four different chromosomal regions were amplified each in at least three tumors. The human orthologs of these common amplified regions are known to be amplified in HCC. All tumors of untreated mice had chromosomal aberrations, including both deletions and amplifications. Amplifications in spontaneous tumors affected fewer chromosomes and were not located preferentially at the chromosomal edges. Comparison of gene expression profiles revealed a significantly enriched expression of oncogenes, chromosomal instability markers and E2F1 targets in the post-PHx compared to spontaneous tumors. Both tumor groups shared the same frequent amplification at chromosome 18. Here, we revealed that one of the regulatory genes encoded by this amplified region, Crem, was over-expressed in the nuclei of murine and human HCC cells *in vivo*, and that it stimulated proliferation of human HCC cells *in vitro*. Our results demonstrate that PHx of a chronically inflamed liver directed tumor development to a discrete pathway characterized by amplification of specific chromosomal regions and expression of specific tumor-promoting genes. Crem is a new candidate HCC oncogene frequently amplified in this model and frequently over-expressed in human HCC.

## INTRODUCTION

Tumor resection and ablation are currently the main treatment options for an early-stage hepatocellular carcinoma (HCC), while tumor recurrence is the main complication following these treatments [1]. Chronic inflammation precedes the majority of HCC cases and significantly contributes to genetic instability [2]. To explore the molecular mechanisms of hepatocarcinogenesis following resection of a chronically inflamed liver in a small animal model, we performed partial hepatectomy (PHx) in the Mdr2-KO mice, a well-characterized model of inflammation-mediated HCC development [3, 4]. Previously, we demonstrated accelerated hepatocarcinogenesis following 35% PHx of either 3-month-old or 9-month-old Mdr2-KO males [5]. We found genomic instability in PHx-induced tumors of the hepatectomized mutants and activation of DNA damage response genes in the non-tumor liver of these mice, probably caused by the regenerative proliferative stress [5]. Now, we used 70% PHx of both male and female Mdr2-KO mice at the age of either 3 or 6 months in order to explore the effects of gender and operation time on accelerated HCC development in this model. In order to explore the specific molecular pathways responsible for the accelerated tumor development, we compared genomic landscapes and gene expression profiles of the PHx-induced and spontaneous HCC tumors developed in this model. We demonstrate that 70% PHx of the chronically inflamed liver induces a specific pattern of chromosomal amplifications in the early HCC tumors and specific patterns of gene expression both in tumors and in the non-tumor liver which are different from those in the non-hepatectomized Mdr2-KO mice. Furthermore, we demonstrate that the gene Crem, which is frequently amplified and over-expressed in the Mdr2-KO HCC, is also frequently over-expressed, although rarely amplified, in human HCC, and stimulates proliferation of human HCC cells.

## RESULTS

### Strain, but not gender, determines the tumor-promoting effect of PHx on HCC development in the Mdr2-KO mice

**Figure 1:**
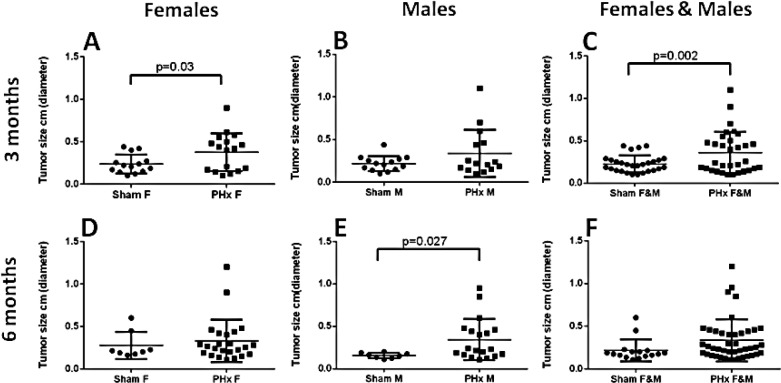
Accelerated HCC development in the Mdr2-KO/FVB mice following 70% PHx Either PHx or sham surgery was performed at the age of either three (A-C) or six (D-F) months; all mice were sacrificed at the age of nine months. Tumor incidence in female (A) or male (B) mice, or females and males together (C) operated at the age of three months (females: PHx n=17, sham n=16; males: PHx n=18, sham n=22). Tumor incidence in female (D) or male (E) mice, or females and males together (F) operated at the age of six months (females: PHx n=25, sham n=10; males: PHx n=20, sham n=11).

In order to delineate the roles of gender and time of PHx in the development of HCC in the chronically inflamed liver, we performed 70% PHx in male and female Mdr2-KO/FVB mice at either three or six months of age; sham operations served as controls (Supplemental Figure 1). PHx significantly increased liver tumor incidence in females operated at the age of three months, and in males operated at the age of six months (Figure 1A,E). For both genders together, the tumor-promoting effect of PHx was statistically significant only when the operation was performed at the age of three months (Figure 1C; Fisher test p=0.002), while, it was only marginally significant when the operation was performed at the age of six months (Figure 1F; Fisher test p=0.067). Tumor load and size were profoundly increased in post-PHx mice: the average tumor load per mouse for both genders together was significantly higher in the post-PHx compared to the sham group at both operation ages, and a proportion of mice having tumors with a diameter larger than 3 mm was two-fold higher in the post-PHx group (Supplemental Table 1A). For both genders, tumors with a diameter larger than 7 mm (for males - larger than 5 mm) were observed only in the PHx-treated mice (Supplemental Table 1B). Remarkably, frequency, size, morphology and developmental stage of the post-PHx liver tumors in Mdr2-KO/FVB mice at the age of nine months, were similar to those of spontaneous tumors observed in the untreated mutants at the age of 13 months (females) or 14 months (males).

To determine the effect of genetic background on the development of HCC in the chronically inflamed liver following PHx, we performed 70% PHx in male Mdr2-KO/B6 mice at six months of age and sacrificed these animals for tumor score at 14 months of age. Surprisingly, there was no significant difference in liver tumor development between the PHx-treated and control groups (Supplemental Figure 2). Both PHx-operated and sham-operated Mdr2-KO/B6 mice at 14 months of age produced a similarly low number of tumors and nodules, compared to the PHx-operated Mdr2-KO/FVB mice at the age of nine months (Figure 1E). Thus, 70% PHx significantly increased liver tumor incidence, load and size in Mdr2-KO mice operated at early ages; however, this tumor-promoting effect was specific for the FVB, but not for the B6 genetic background.

### Comparison of cell proliferation and DNA damage markers in non-tumor and tumor liver tissues between post-PHx and untreated Mdr2-KO/FVB mice

**Figure 2:**
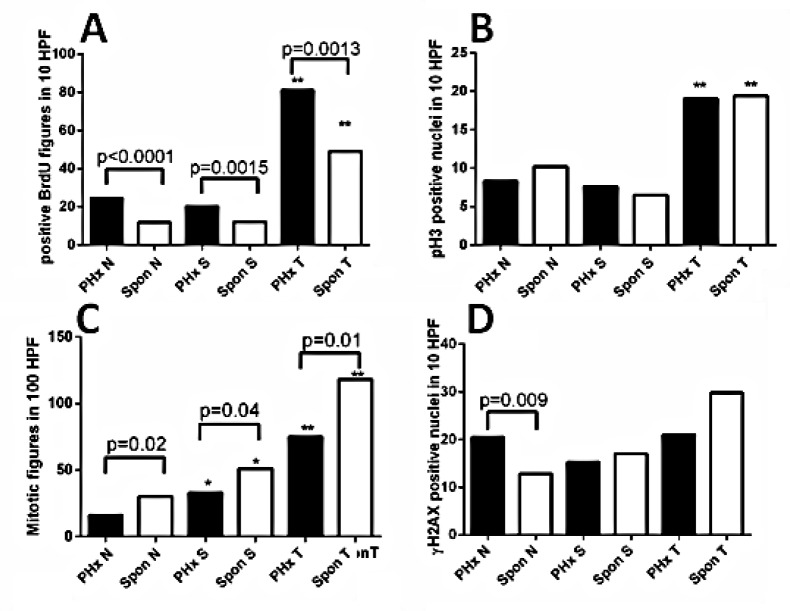
Quantification of proliferation and DNA damage markers in the liver of hepatectomized and untreated Mdr2-KO/FVB mice Comparative quantification of the appropriate markers in the distant non-tumor (N), surrounding non-tumor (S) and tumor (T) liver tissues from 9-month-old post-PHx (PHx) and 13-14-month-old untreated (Spon) Mdr2-KO mice. The representative IHC pictures are shown in Supplemental Figure 3. Frequency of hepatocytes positive for: (A) BrdU; (B) phospho-histone-3; (C) mitotic figures; (D) γH2AX. *, statistical significance between the surrounding and non-tumorous tissues of the same group (*t*test p≤0.002); **, statistical significance between tumors and non-tumor/surrounding tissues (*t*test p≤0.001). Six mice per each group. HPF: high power field.

To explore the mechanisms of the accelerated HCC development by PHx, we compared liver tumors and their matched non-tumor liver tissues between 9-month-old hepatectomized and 13-14-month-old untreated Mdr2-KO mice. From each experimental group, we selected six well differentiated HCC tumors similar in size range and morphology between groups. All tumors were well protruding from the liver and contained at least 90% of tumor cells on histological sections. We compared cell proliferation and DNA damage markers in tumors and their adjacent non-tumor (surrounding) as well as distant non-tumor (non-tumor) liver tissues between both experimental groups. BrdU inclusion in hepatocytes for all three tissue types was significantly higher in the post-PHx compared to the untreated (spontaneous) group (Figure 2A, Supplemental Figure 3A-D). However, there was no difference in the level of phosphorylated histone 3, a marker of the mitotic M phase, between groups (Figure 2B, Supplemental Figure 3E-H), while there were decreased numbers of mitotic figures in the post-PHx compared to the untreated group in all tissue types (Figure 2C, Supplemental Figure 3I-L). Immunostaining revealed no difference in nuclear p21 level between non-tumor liver tissues of experimental groups (not shown). TUNEL assay revealed similar and very low levels of positive cells in both post-PHx and untreated non-tumor liver (not shown), while the level of the phosphorylated histone 2AX, a marker of either DNA damage, or stalked replication forks, was significantly higher in the post-PHx non-tumor liver (Figure 2D, Supplemental Figure 3M-P). These results are consistent with the increased DNA repair process in the non-tumor and tumor liver tissues, and stalked replication forks in the non-tumor liver tissue of hepatectomized compared to the untreated Mdr2-KO/FVB mice.

### Specific genomic amplifications in PHx-induced liver tumors

**Figure 3:**
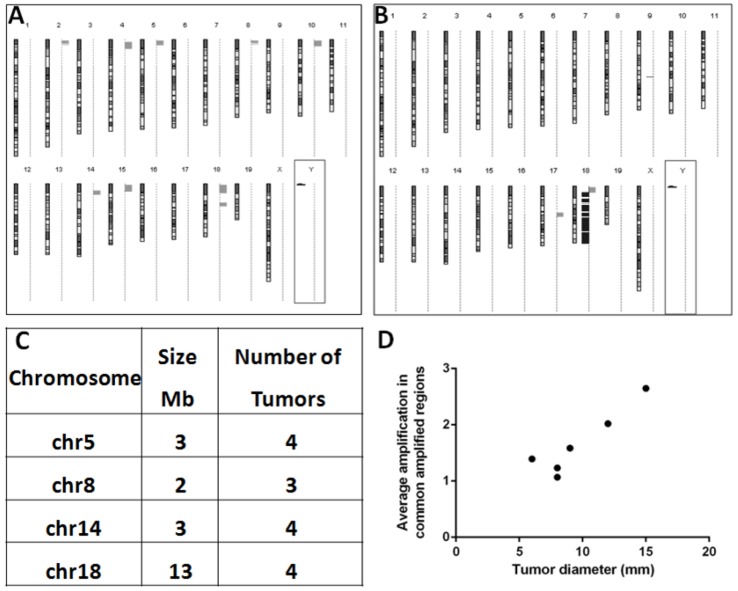
Specific pattern of genomic amplifications observed in the post-PHx tumors of Mdr2-KO/FVB mice (A, B) Typical profiles of chromosomal aberrations in either a post-PHx tumor (A), containing only amplifications (gray bars on the right of each chromosome), or a spontaneous tumor (B), containing both amplifications and deletions (black bars on the right of each chromosome); centromeres - at the top of each chromosome. (C) Four genomic regions that were frequently amplified in the post-PHx tumors. (D) In post-PHx tumors, the average amplification level positively correlated with tumor size. Axis “Y” - average amplification level (fold-change) of common amplified regions per tumor; axis “X” - tumor diameter, mm. Pearson correlation r=0.9234, p=0.0086.

To understand the molecular mechanisms of the accelerated tumor development in post-PHx mice, we compared, using aCGH, genomic aberrations in six tumors from each of the experimental groups described above (Supplemental Figure 4; aCGH maps of four among six post-PHx tumors were published by us previously [5]). Among PHx-induced tumors, 5 of 6 had major chromosomal aberrations, and all of them were amplifications affecting multiple chromosomes (Figure 3A). Most amplifications were located near the acrocentric centromeres of murine chromosomes. Four different chromosomal regions (size 2 to 13 Mb) were amplified (1.3 to 12.3 fold) each in at least three tumors (Figure 3C). The frequency of amplifications of common amplified regions correlated with tumor size (R^2^= 0.8527; Figure 3D). All six spontaneous tumors developed by untreated Mdr2-KO mice had chromosomal aberrations, including both deletions (4/6 tumors) and amplifications (5/6 tumors). Amplifications in spontaneous tumors affected significantly less chromosomes and were not located preferentially at chromosomal edges. PHx-induced and spontaneous tumors shared the same frequent amplification only at chromosome 18 (at least 3 samples in each group; Figure 3A,B; Supplemental Table 2). Bioinformatic comparison of the genomic data of post-PHx tumors from Mdr2-KO mice with 76 tumors from three different HCC mouse models (Supplemental Figure 5), and with 155 human HCCs (compared by synteny; Supplemental Figure 6) revealed only rare overlaps of genome amplification patterns between the PHx-induced tumors and other murine or human HCCs. Nevertheless, analysis of the published literature demonstrated that many human chromosomal regions syntenic to the four murine frequently amplified regions were amplified in some human HCC tumors (Supplemental Table 3).

### Specific patterns of gene expression in the PHx-induced tumors

Genome-scale gene expression analysis of the PHx-induced tumors and their distant non-tumor liver tissues using PCA (Supplemental Figure 7) and hierarchical clustering (Supplemental Figure 8) revealed that most non-tumor samples clustered together. Two female tumors produced a separate cluster, while four male tumors did not; age at PHx and the number and level of amplifications did not affect clustering. The distance between the matched tumor and non-tumor samples in both of these analyses directly correlated with the total number of aberrantly expressed genes in the tumor (Supplemental Table 4).

**Figure 4:**
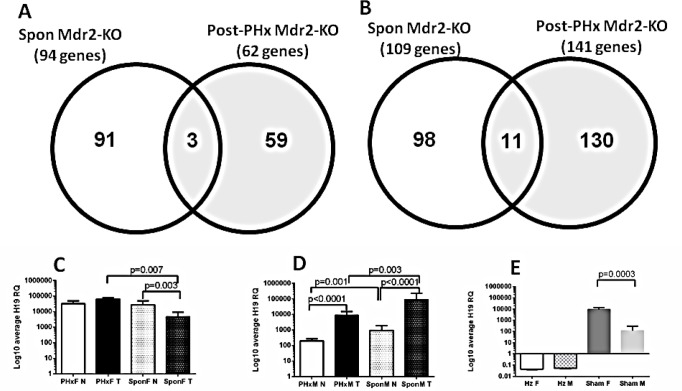
Gene expression profiling of the post-PHx and spontaneous tumors from Mdr2-KO mice (A, B) Venn diagram, comparing numbers of differentially expressed liver genes (tumor/non-tumor) in the 9-month-old post-PHx mice with those of untreated 16-month-old mice; six pairs of tumors and their matched non-tumor liver tissues from each group. (A) Genes down-regulated in at least four of six tumors below the threshold of −1.8-fold-change. (B) Genes up-regulated in at least three of six tumors above the threshold of 1.8-fold-change. (C-E) H19 expression (qRT-PCR) in the distant non-tumor (N) and tumor (T) liver tissues from 9-month-old post-PHx (PHx) and 13-14-month-old untreated (Spon) Mdr2-KO females (C) and males (D), or in 9-month-old controls (E): Hz, Mdr2-heterozygotes; F or M - sham-operated Mdr2-KO females or males. Four-to-five mice per group; statistics - using the F-test.

Comparison of the genome-scale gene expression profiles of these six post-PHx tumors with those of spontaneous tumors of the 16-month-old Mdr2-KO mice which were analyzed previously [4] revealed surprisingly few common down-regulated (Figure 4A) and up-regulated (Figure 4B) genes in both tested datasets. We compared both datasets also for enrichment in four cancer-associated gene expression signatures: up-regulation of HCC-specific oncogenes, or chromosomal instability (CIN) genes, or E2F1 targets, and down-regulation of HCC-specific tumor suppressors (Table 1) [6, 7]. At least 50% of post-PHx tumors were significantly enriched in Oncogenic, CIN, and E2F1 gene expression signatures, while in spontaneous tumors only solitary tumors were significantly enriched in either Oncogenic or E2F1 signature. For both groups, only solitary tumors were significantly enriched in the tumor suppressor signature.

**Table 1:** Gene expression signatures in the PHx-induced and spontaneous HCC tumors of Mdr2-KO/FVB mice The PHx-induced tumors as compared to spontaneous tumors were enriched with the “Oncogene”, “CIN” and “E2F1”, but not with “Tumor suppressors”, signatures.

	Tumor ID	Tumor suppressors	Oncogenes	CIN signature genes	E2F1 signature genes
**Fold change threshold^a^:**		**≤ −1.5**	**≥ 1.5**	**≥ 1.8**	**≥ 1.8**
**Mdr2-KO mice following PHx 9-month-old**	PHx2F	0.59	**0.0216**	**1.06E-07**	**2.89E-09**
	PHx4M	1	**0.0498**	**4.53E-12**	**8.13E-08**
	PHx3M	0.77	0.11	1	0.09
	PHx1M	1	0.46	0.15	1
	PHx5M	0.63	0.32	**0.0058**	0.06
	PHx6F	**0.0384**	**0.0269**	**8.95E-09**	**8.07E-07**
**Mdr2-KO mice untreated 16-month-old**	49-T1	0.82	1	1	1
	84-T1	**0.0270**	1	0.09	**3.51E-02**
	93-T1	1	**5.61E-05**	0.17	0.65
	93-T2	0.11	1	1	0.19
	96-T1	0.19	1	1	0.73
	96-T2	0.82	0.58	1	0.54

a Fold change thresholds in tumors vs. matched non-tumorous tissue.

Gene lists for analysis of expression signatures of oncogenes, tumor suppressors and E2F1 targets have been described by us previously [7]; the 25-gene CIN signature – as described in [6].

Statistical significance (p value) of each gene signature was determined by Fisher test using R-software. Bold numbers indicate p-value < 0.05.

Genome-scale gene expression profiling of the six PHx-induced tumors has been performed in the current study, and profiling of the six spontaneous tumors from untreated Mdr2-KO mice – in our previous study [4].

The microarray data demonstrated a very high expression of the H19 transcript in the non-tumor liver of hepatectomized females. The H19 transcript is known to be frequently highly expressed in HCC [8]. We confirmed this result and explored the effect of gender and PHx on H19 expression using qRT-PCR of tumor and non-tumor RNA samples from untreated (13-14-month-old) and post-PHx (9-month-old) livers from Mdr2-KO males and females. There was a prominent gender disparity in the H19 expression in tumor *versus* non-tumor liver: in females, it was similar in the post-PHx tumors, while it significantly decreased in spontaneous tumors (Figure 4C), whereas in males, it was significantly increased in both spontaneous and post-PHx groups (Figure 4D). Altogether, gene expression data indicate that hepatocarcinogenesis differed significantly between the post-PHx and spontaneous tumors of Mdr2-KO/FVB mice.

### Correlation between gene amplification and expression in the PHx-induced tumors

There was a significant correlation between genomic amplification and elevation of gene expression in all amplified regions of the PHx-induced tumors (Supplemental Table 5). Furthermore, while the observed average ratio between the up-regulated and down-regulated genes in all PHx-induced tumors was about 50%, in the common amplified regions, 95% of differentially expressed genes were up-regulated. Correlation between the amplification and expression levels was confirmed by semi-qRT-PCR for 22 genes in common amplified regions (Supplemental Table 6). The best correlation with gene expression was found for genomic amplifications over 2.5-fold (Supplemental Figure 9), similar to what was described previously for breast cancer tumors [9].

### Pathway analysis of genes in the common amplified regions of the post-PHx tumors

The common amplified regions of the post-PHx tumors contained 122 genes; 32 among them were known in the literature as having a role in cancer. Analysis of signaling pathways for these 122 genes by GO, revealed a significant prevalence of the Wnt signaling pathway (Supplemental Table 7). However, immunostaining for beta-catenin revealed only rare scattered positive hepatocyte nuclei and variable cytoplasmic staining in both post-PHx and spontaneous tumors (Supplemental Figure 10). The only post-PHx tumor sample which did not contain amplifications (PHx5M) had a typical gene expression signature of the activated Wnt signaling (Supplemental Table 8), but not beta-catenin-positive nuclear immunostaining (not shown).

### Gene signatures of the early HCC development/recurrence in tumor and non-tumor liver tissues of the post-PHx Mdr2-KO/FVB mice

**Figure 5:**
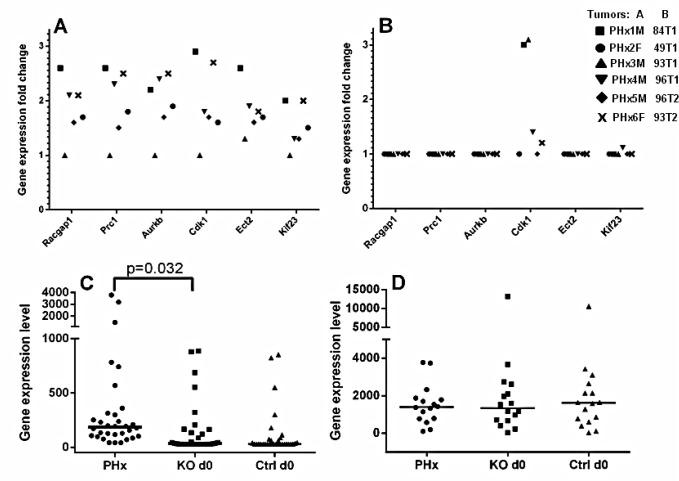
Post-PHx tumors and their matched non-tumor liver tissues of Mdr2-KO mice have prominent “poor prognosis in HCC” signatures (A-B) Comparison of the “tumor/non-tumor tissue” gene expression signatures of Racgap1-interacting genes (axis “Y” – fold-change ratio, microarray data) between the six tumors from 9-month-old post-PHx Mdr2-KO mice (A) and the six tumors from untreated 16-month-old Mdr2-KO mice (B). (C-D) Comparison of “non-tumor poor prognosis in HCC signatures” between non-tumor livers from the following groups of 9-month-old mice: four Mdr2-KO post-PHx (PHx), four untreated Mdr2-KO (KO d0), and three untreated Mdr2-heterozygous controls (Ctrl d0). Axis “Y” - absolute gene expression levels, microarray data. (C) Genes of the “non-tumor poor prognosis in HCC signature”. (D) A set of 16 housekeeping genes used for normalization of two datasets.

To explore the relevance of this model (PHx of the chronically inflamed murine liver) to the process of HCC development/recurrence in patients, we analyzed the significance of the known appropriate gene expression signatures in the tumor and non-tumor samples of the post-PHx Mdr2-KO/FVB mice. Recently, it was shown that in tumors removed by curative surgery, up-regulation of the Racgap1 together with several other genes of its interactome is associated with the early HCC recurrence [10]. Here we show that five of the six post-PHx tumors had a similar co-upregulation pattern for most of Racgap1-associated genes (Figure 5A), while none of the spontaneous tumors from 16-month-old Mdr2-KO/FVB mice had this expression signature (Figure 5B). A prominent prognostic expression signature of 186 genes wherein the over-expression of its specific subsets was correlated with either poor or good prognosis, has been confirmed recently as having a prognostic significance for HCC development based on the expression profile of the early-stage cirrhotic liver samples [11]. We next compared the expression of the top 40 genes which were over-expressed in the “poor prognosis” signature between non-tumor liver tissues of the post-PHx males obtained in the current study, and the age-matched untreated Mdr2-KO/FVB and control liver samples that were studied by us previously [5]. Among the tested 40 genes, 31 were presented in both microarrays, and 22 of them were up-regulated in the post-PHx compared to the untreated Mdr2-KO livers (Figure 5C). Expression of the most of 16 housekeeping genes, which were used for normalization of expression levels in both comparative datasets, was similar between experimental groups (Figure 5D). Overall, the human “poor prognosis” HCC signatures were significantly more prominent in the post-PHx compared to the untreated Mdr2-KO/FVB liver indicating that PHx of these mice increases the expression of the HCC development/recurrency markers in both tumor and non-tumor liver tissues even several months following the operation.

### Crem is a new candidate HCC oncogene which is frequently amplified and over-expressed following PHx

The most common amplified region among both post-PHx and spontaneous tumors was the one on chromosome 18, containing 22 unique genes: it was amplified in 4/6 post PHx tumors and in 3/6 spontaneous tumors (Figure 3C, Supplemental Table 2). We focused on the gene Crem encoded by this region due to its involvement in cancer [12, 13], including HCC metastasis [14], LR following PHx [15], and circadian regulation [16]. Using semi-qRT-PCR of the matched tumor and non-tumor samples, we confirmed that Crem was over-expressed (FC>=1.8) in all tumor samples where it was amplified (FC >= 1.4) (Supplemental Table 6, Supplemental Figure 11). The correlation between Crem genomic amplification and transcript expression was significantly higher in post-PHx compared to spontaneous tumors (Supp Figure 11A,B).

**Figure 6:**
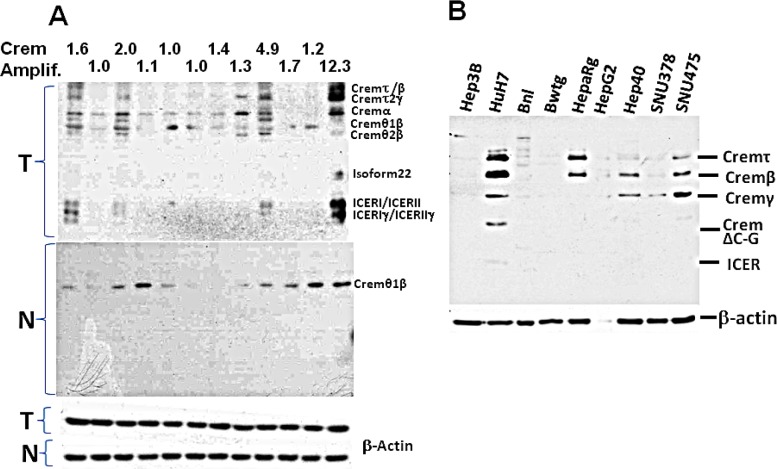
Expression of the Crem protein isoforms in murine liver at tumor stage and in human and murine HCC cells Immunoblotting of the Crem protein in murine HCC tumors (A) and in human and murine HCC cell lines (B); Crem isoforms are designated on the right. In (A): upper panel (T) – 12 murine tumors (6 post-PHx and 6 spontaneous) with the *Crem* amplification numbers above (tumor/non-tumor, based on aCGH); middle panel (N) – their matched non-tumor liver samples; bottom panel – beta-actin.

**Figure 7:**
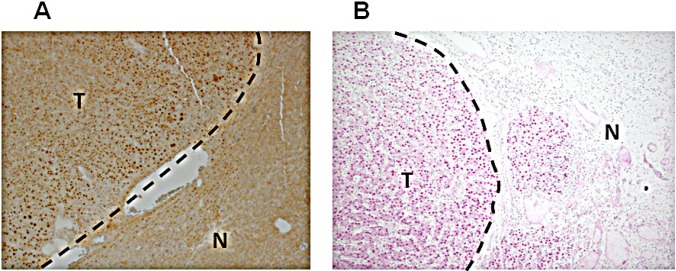
Increased Crem protein expression in the nuclei of HCC tumor cells Representative images of IHC staining of the Crem protein in murine (A) and human (B) HCC (T) and adjacent non-tumor liver (N). Magnification x100.

Crem has multiple splicing isoforms (Supplemental Table 9). Using immunoblotting, we revealed that more Crem protein isoforms and at higher levels were expressed in tumors than in matched non-tumor liver tissues, reaching the highest levels in tumors having genomic Crem amplifications (FC>1.5; Figure 6A). Immunostaining of paraffin-embedded murine liver tissues revealed strong Crem staining in hepatocyte nuclei of tumors containing Crem amplifications compared to the surrounding non-tumor tissues (Figure 7A). We have tested CREM protein expression in 13 human HCC samples of various etiologies, and have found CREM-positive nuclei in 11 HCCs (85%), but not in the non-tumor cirrhotic liver (Table 2, Figure 7B).

**Table 2:** Characteristics of the HCC patients and their tumors

Patient	CREM^1^	Age	Gender	Etiology	Cirrhosis^2^	Grading	T-Status
1	Negative	71	Male	Hepatitis C	Yes	2	1
2	Positive	56	Male	Crypotgenic	Yes	1	2
3	Negative	60	Male	Alcohol	Yes	2	2
4	Positive	75	Male	Cryptogenic	No	2	1
5	Positive	62	Male	Hepatitis C	Yes	2	3
6	Positive	60	Male	Hepatitis C, alcohol	Yes	2	3b
7	Positive	55	Female	Hepatitis C	Yes	2	3a
8	Positive	67	Male	NASH	No	3	3a
9	Positive	65	Male	Hepatitis C	Yes	1	1
10	Positive	58	Female	Cryptogenic	No	2	2
11	Positive	30	Female	Adenoma	No	2	3a
12	Positive	76	Male	Hemochormatosis	No	2	2
13	Positive	67	Male	Hepatitis B	No	2	1

1 Results of the IHC staining of tumor samples for CREM. Positive CREM-staining in the tumor was more intense at the borders.

2 In patients with cirrhosis (1, 2, 3, 5, 6, 7, 9) no CREM-staining was observed in the non-tumor liver tissue.

**Figure 8:**
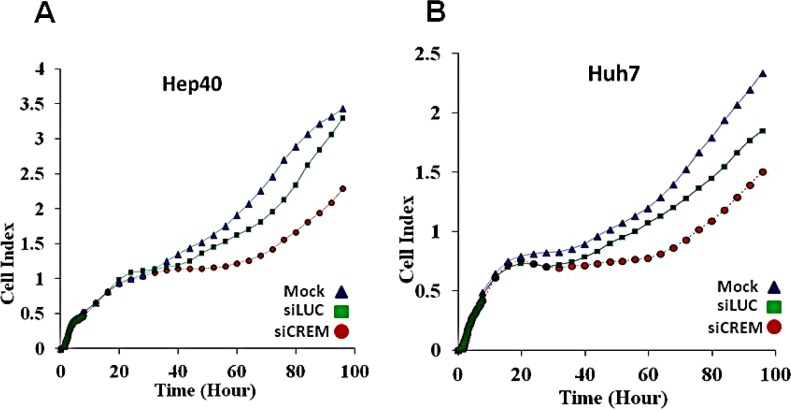
Inhibitory effect of the Crem protein knockdown on proliferation of HCC cells Reduced proliferation rate of two tested human HCC cell lines following CREM knockdown by siRNA: mock-treated (blue rectangles), or siLuc-treated (green squares), or siCREM-treated (red circles) Hep40 (A) and Huh7 (B) cells (*t*test p=0.01 for both cell lines when compared siCREM to siLuc).

Several Crem isoforms were detected by immunoblotting in five out of nine tested human and mouse HCC cell lines (Figure 6B). Human lines Huh7 and Hep40 were selected for studying the role of CREM in cancer cell proliferation by knockdown experiments using CREM-specific siRNA. Immunoblotting revealed that the selected CREM-specific siRNAs efficiently decreased protein levels of the most CREM isoforms, especially ICER (Supp Figure 11D). The xCELLigence proliferation assay demonstrated a significant decrease of the cell proliferation rate following transient transfection of both Huh7 and Hep40 cell lines with CREM-specific siRNA, suggesting that CREM is important for the proliferation of these HCC cells (Figure 8). Importantly, no significant effect of this transfection on cell size was observed (Supp Figure 11E). These results demonstrate that gene Crem, frequently amplified in both post-PHx and spontaneous tumors of the Mdr2-KO mice, is expressed in hepatocyte nuclei in correlation with its genomic amplification in murine HCC tumors; human CREM is expressed in the tumor cell nuclei of the majority of evaluated HCCs, and is required for the efficient proliferation of human HCC cell lines *in vitro*.

## DISCUSSION

### Strain specificity of the PHx-induced tumors in the Mdr2-KO liver suggests a direct correlation between the degree of chronic liver inflammation and a number of tumor cell precursors at a young age

We have revealed strain-specificity of the effect of PHx on tumor acceleration in Mdr2-KO males operated at the age of six months: in the FVB/N strain, the tumor-promoting effect of PHx could be already detected at the age of nine months, whereas in the B6 strain, no effect of PHx on HCC development could be observed even at the age of 14 months. This result correlates with our previous findings of a reduced inflammation at the early age and retarded tumor development at the adult age in the Mdr2-KO/B6 compared to the Mdr2-KO/FVB strain [17]. Importantly, 70% PHx of B6 males does accelerate tumor appearance in chemically-induced hepatocarcinogenesis [18]. In our previous work we suggested that genomic unstable hepatocytes generated during chronic inflammation are the tumor-initiating cells in this model, which escape cell cycle arrest and re-enter cell cycle due to PHx-induced regenerative proliferative stress [5]. We suggest that in the chemically-induced carcinogenesis and in the highly inflamed Mdr2-KO/FVB strain, such potentially tumorigenic cells appear early in the liver, and thus PHx accelerates tumor development. In the Mdr2-KO/B6 males, chronic liver inflammation decreases significantly after two months of age, probably blocking the formation of the tumor-initiating cells at least till the age of six months; thus, PHx in these mice at this age did not have a tumor-promoting effect. This probably explains also the gender difference in the Mdr2-KO/FVB model: the tumor-promoting effect of PHx in females was significant already at the age of three months, while in males – mainly at six months. The Mdr2-KO/FVB females are more inflamed at the early ages than males (our unpublished data) and thus, probably generate the tumor-initiating cells in the liver earlier. Interestingly, inflammation-mediated HCC develops in aged Mdr2-KO/FVB females earlier than in males (see “Results”), whereas spontaneous HCC develops in the parental FVB/NJ strain in old males only [19].

### Potential mechanisms of the observed genome amplifications

Recently, it was demonstrated that HCCs from Mdr2-KO/FVB mice are similar to pediatric HCCs of similar etiology in terms of low frequency of point mutations and high frequency of gene amplifications [20]. In contrast to our findings, the authors could detect only a negligible number of deletions in spontaneous tumors from Mdr2-KO/FVB mice. This discrepancy could arise from a different methodology: we used the direct aCGH method, whereas Iannelli et al. used a non-direct method for the detection of chromosomal aberrations in mice [20]. The existence of deletions in spontaneous tumors of Mdr2-KO/FVB mice was also reported by us previously [4]. A striking difference in the patterns of chromosomal aberrations between PHx-induced and spontaneous tumors that we found here, raises a question on the potential mechanism of PHx-induced specific genomic amplifications without deletions (a probability to observe six tumors without deletions by chance here is 0.0014). The recent finding of genomic amplifications of the Kras oncogene in the early lung tumors appearing in the FVB/N, but not in the mixed genetic background [21], indicates that this “amplification pattern” may be also strain-specific. However, gene amplification by nonhomologous subtelomeric exchanges has been documented even for the WT murine species [22], and centromeres are known hotspots of mitotic recombination [23]. Replication stress is a prominent pathway to genomic instability [24]; loss of DNA replication control may cause inappropriate re-replication resulting in gene amplification [25]. Our analysis of the cell proliferation and DNA damage markers (Figure 2 and Supplemental Figure 3) indicates that an increased replication stress and DNA repair in the post-PHx liver of Mdr2-KO/FVB mice could be responsible for the increased CIN in the PHx-induced tumors, in agreement with our previous findings in mice [5]. The aCGH method that we used provides only a relative quantification of different genomic regions, and not locations of the amplified genes: whether they are extra-, intra- or inter-chromosomal.

### Functional analysis of the genes that were frequently amplified in PHx-induced tumors

Determination of causative cancer genes in chromosomal aberrations is complicated by the known examples of cooperation between both co-amplified oncogenes [26] and co-deleted tumor suppressors [27]. PHx-induced tumors had a significantly higher proportion of common up-regulated genes (69.5%) compared to spontaneous tumors of untreated Mdr2-KO/FVB mice (53.7%), whereas the total number of common aberrantly expressed genes in each group was identical (according to the parameters described in Figure 4). These up-regulated genes included HCC-specific oncogenes (they are listed in our recent work [7]), E2F1 and CIN signature genes [6]. Remarkably, CIN and E2F1 genes significantly overlap, as most CIN genes are E2F1 targets [7]. Although GO analysis of the 122 genes from the common amplified regions revealed a prevalence of the Wnt signaling pathway, IHC demonstrated that all PHx-induced tumors either did not have, or had only a rare and fragmentary β-catenin-positive nuclear staining.

### Other gene expression signatures that characterize the PHx-induced tumors

Prediction of tumor recurrence which jeopardizes survival of about 70% of HCC patients within 5 years following tumor resection or ablation is of high clinical importance [28]. Several gene expression signatures of the resected tumor and/or non-tumor liver tissue that may predict early HCC development/recurrence either alone [10, 11], or in combination with clinical and pathological data [28] were recently developed. Our finding that both tumor-specific [10] and non-tumor-specific [11] “poor prognosis” signatures were significantly more prominent in the liver of hepatectomized compared to untreated Mdr2-KO/FVB mice indicates that these mice may serve as a model for human HCC development/recurrence.

Validation of H19 expression on an expanded set of liver samples by qRT-PCR revealed different expression patterns between PHx-induced and spontaneous tumors for Mdr2-KO/FVB females, but not for males (Figure 4C-E). The H19 expression is known to be frequently up-regulated in cancer including HCC [8], acting in different models either as a tumor suppressor [29], or as an oncogene [30]; its role in hepatocarcinogenesis is still debated. Our findings of a prominent gender disparity in the relative tumor/non-tumor H19 expression in both naïve and hepatectomized Mdr2-KO/FVB mice and of a high H19 expression in the non-tumor liver tissue of Mdr2-KO/FVB females may indicate that H19 has different functions in hepatocarcinogenesis of each gender.

### Crem is a new candidate HCC oncogene

In our search for candidate cancer-causing genes in frequently amplified regions, we focused on the gene Crem because it was amplified in 4/6 post PHx tumors and in 3/6 spontaneous tumors, and also due to its involvement in cancer [12, 13], including HCC metastasis [14], LR following PHx [15], circadian regulation [16, 31] and cytokine production [32]. Remarkably, murine liver tumors containing Crem amplifications demonstrated high nuclear Crem expression (Figure 7A) and more expressed Crem protein isoforms compared to the matched non-tumor liver (Figure 6A). In human HCC gene expression datasets, CREM was infrequently up-regulated in tumors (although frequently up-regulated in cirrhosis); similarly, CREM DNA was infrequently amplified (Supplemental Table 3, 10p11.21 region). Nevertheless, IHC demonstrated CREM-positive nuclear staining in 11/13 tested human HCC tumors, but not in the surrounding non-tumor liver, independently on the patient's age and gender and on HCC etiology (Table 2), probably indicating a transcript level-independent mechanism of CREM up-regulation. CREM knock-down experiments *in vitro* revealed its pro-proliferation role in two human HCC cell lines, thus demonstrating that CREM is a new candidate HCC oncogene.

In summary, we demonstrated that PHx of the chronically inflamed murine liver accelerated HCC development by a specific pathway characterized by amplification of chromosomal regions located near the acrocentric centromeres of murine chromosomes and by expression of specific tumor-promoting genes, including those of the “poor prognosis” HCC signatures. We also identified Crem as a new candidate HCC oncogene frequently amplified and up-regulated in this murine HCC model and frequently over-expressed in human HCC.

## MATERIALS AND METHODS

Full details are available in the Supplemental Material

### Mice

All animals received human care. All animal experiments were performed according to national regulations and guidelines of the Institutional Animal Welfare Committee (NIH approval number OPRR-A01-5011). The FVB.129P2-Abcb4^tm1Bor^ (Mdr2-KO/FVB) and the C57Bl/6 Mdr2-KO (Mdr2-KO/B6) mice were described previously [17]. The 70% PHx or sham surgery was performed as described [5].

### RNA analysis

Total RNA was isolated from frozen liver tissues and subjected to gene expression profiling using GeneChip Mouse Gene 1.0 ST Array (Affymetrix, Santa Clara, CA) as described [4]. Data were analyzed by GCRMA preprocessing algorithm using the Partek software (ProSoftware, Partek, St Louis, MO, USA). For validation of gene expression by RT–PCR, reverse transcription of total RNA was performed using the qScript^TM^ cDNA Synthesis Kit (#95047) and Perfecta Sybr Green Fast Mix ROX (#95073) (both from Quanta BioSciences Inc., Gaithersburg, MD, USA). The quantitative PCR assay was performed on an AB 7900 HT fast real-time PCR system (Applied Biosystems, Foster City, CA, USA) or CFX384^TM^ Real-Time System with C1000 Touch Thermal Cycle (BioRad, Hercules, CA, USA). The semi-quantitative RT-PCR and the primers used for RT-PCR are described in the Supplemental Materials and Methods.

### DNA analysis

Total DNA was isolated from frozen tumors and their matched non-tumor liver tissues as described [4] and subjected to array-based comparative genomic hybridization (aCGH) analysis using Mouse Genome CGH Microarrays 4×44K (Agilent, Santa Clara, CA). Data for both genome and transcriptome microarray analyses can be accessed from the GEO-NCBI database repository (GSE61427).

### Protein analysis

Protein detection by immunoblotting and immunohistochemial staining was performed as described in the Supplemental Materials and Methods.

### Statistical analysis and calculation software

All the *in vitro* experiments were performed in triplicates, excluding the cell proliferation rate assay using a xCELLigence system, which was performed in duplicates; in the *in vivo* experiments, at least 3 mice per group were used. All parameters were evaluated with the two-tailed *t*-test. Statistical evaluation of differential expression between the experimental groups was performed using the ANOVA test. A p-value of 0.05 or less was considered significant. *t*-test, correlation test, and f-test were performed using the GraphPad Prism version 6.04 for Windows (GraphPad Software, La Jolla California USA, www.graphpad.com). Stained hepatocytes IHC sections were counted by the NIS-Elements Microscope Imaging Software (Nikon Instruments Inc.1300 Walt Whitman Road, Melville, NY, U.S.A). The results of protein immunoblotting and semi-quantitative RT-PCR were calculated using ImageJ (Image processing and analysis in Java, NIH, U.S.A). To evaluate the effect of PHx on tumor incidence, the Fisher exact test was calculated online (http://www.quantpsy.org/fisher/fisher.htm). The Pearson's correlation coefficient (Supplemental Table 5) was calculated for all 1,256 genes with matching copy number and expression profiles. The distribution of correlation values was compared to a randomized background distribution and was found to be significantly enriched with positive correlation values (p<E-33). The randomized background distribution was generated by repeatedly (100 times) shuffling the samples in the expression dataset only and recalculating the non-sample matched correlation values. The disparity between the two distributions was estimated using the Kolmogorov-Smirnov test.

## SUPPLEMENTARY MATERIAL FIGURES AND TABLES



## List of Abbreviations

Abcb4 (Mdr2): adenosine triphosphate–binding cassette b4 (multi-drug resistance 2)
aCGH: array-based comparative genomic hybridization
B6: C57Black/6 strain
BrdU: bromodeoxyuridine
CIN: Chromosomal Instability
Crem: cAMP responsive element modulator
FC: Fold Change
FVB: Friend virus B-type/N strain
GO: Gene Ontology
GCRMA: GeneChip robust multiarray analysis
HCC: hepatocellular carcinoma
ICER: Inducible CAMP Early Repressor
IHC: immunohistochemistry
LR: liver regeneration
KO: knockout
mRNA: messenger RNA
PHx: Partial hepatectomy
PCA: Principal Component Analysis
qRT-PCR: quantitative RT-PCR
RT-PCR: reverse transcription polymerase chain reaction
TUNEL: Terminal deoxynucleotidyl transferase dUTP Nick End Labeling
Wnt: Wingless-Type MMTV Integration Site Family
WT: wild type
