# Supplementary material for: Specific genomic and transcriptomic aberrations in tumors induced by partial hepatectomy of a chronically inflamed murine liver
Source: Oncotarget. 2014 Sep 25;5(21):10318–31. doi: 10.18632/oncotarget.2515 (PMC4279375; doi:10.18632/oncotarget.2515)
Supplement: Supplementary file 1 [file oncotarget-05-10318-s001.pdf]

Specific genomic and transcriptomic aberrations in tumors induced by partial hepatectomy of a chronically inflamed murine liver

Supplementary Material

Operations with mice

All mice were fed a regular diet with drinking water *ad libitum* and under controlled SPF conditions (22°C, 55% humidity, and 12-hour day-night rhythm). Seventy percent partial hepatectomy (PHx) or sham surgery was performed on either 3-month-old or 6-month-old Mdr2-KO mice as described (1, 2). The surgical procedures were performed under sterile conditions, using ketamine and xylasine anesthesia; operations were performed between 09:00 and 12:00 hours AM. At the terminal time points, mice were anesthetized using isoflurane and sacrificed by cervical dislocation. Blood was collected by orbital bleeding. Livers were rapidly excised and weighed; part of the left lobe was fixed in 4% buffered formaldehyde for histological analysis, and the remaining liver tissue was quickly frozen in liquid nitrogen and stored at -80°C. All experiments were approved by The Hebrew University Ethical Committee for Animal Experimentation.

Array-based Comparative Genomic Hybridization (aCGH)

Genomic aberrations in the liver DNA were determined using aCGH on Mouse Genome CGH Microarrays 4x44K (Agilent, Santa Clara, CA) by Miltenyi Biotec GmbH (Bergisch Gladbach, Germany). DNA samples purified from murine HCC tumors and their matched distant non-tumor liver tissues were subjected to digestion by AluI and RsaI restriction endonucleases and used as templates for a genomic DNA labeling reaction using the Genomic Enzymatic Labeling Kit (Agilent Technologies) according to the manufacturer’s protocol. The yields of labeled DNA and the dye-incorporation rate after Klenow labeling were determined with the ND-1000 Spectrophotometer (NanoDrop Technologies). The hybridization procedure was performed according to the Agilent Oligonucleotide Array-Based CGH for Genomic DNA Analysis protocol V5.0 using the Agilent Oligo aCGH Hybridization Kit (Agilent

Technologies). The corresponding Cy3- and Cy5-labeled DNA were combined and hybridized at 65°C 24 hours to an Agilent Mouse Genome CGH Microarray 4x44K, using Agilent's recommended hybridization chamber and oven. Subsequently, the microarrays were washed once with Agilent Oligo aCGH Wash Buffer 1 at room temperature for 5 minutes followed by a second wash with Agilent Oligo aCGH Wash Buffer 2 at 37 °C for one minute. Finally, the microarray was washed once with acetonitril for one minute at room temperature and slowly removed from the acetonitril solution to let it dry at room temperature. Fluorescence signals of the hybridized Agilent Mouse Genome CGH Microarrays were detected using Agilent's DNA microarray scanner (Agilent Technologies).

The Agilent Feature Extraction Software (FES) was used to read out and process the microarray image file. The software determines feature intensities and ratios (including background subtraction and normalization), rejects outliers, and calculates statistical confidences (p-values). Further analysis and visualization of the hybridization results was performed with the Agilent CGH Analytics software (V 3.5). Here, the following aberration filter settings have been used:

(i) Minimum number of probes present in an aberrant region = 2

(ii) Minimum absolute average log2 ratio for region = 0.4 corresponds to a fold change of 1.32.

(iii) The statistical analysis of aberrant regions was based on the aberration detection method ADM-2.

RNA purification and cDNA synthesis

Total RNA was isolated from frozen liver tissues with Trizol reagent (Invitrogen, Carlabad, CA, USA) followed by twice Phenol-Chlorophorm extraction and isopropanol precipitation. The cDNA was obtained from 1 µg total liver RNA. Twelve units of the RNasine RNase inhibitor and 1/25ul random hexamer primers (Promega, WI, USA) in DEPC-treated water were preheated at 90 ^{o}C for 1 min to denature secondary structures. The mixture was then cooled gradually (0.2 ^{o}C/S to 45°C and then 5 µl 5X RT Buffer, and 0.5 mM dNTPs (Deoxynucleotide triphosphate set, Roche, Indianapolis, IN, USA)) with 100 units Moloney murine leukemia virus reverse transcriptase enzyme (Promega, WI, USA) added for a

total volume of 25 µl. The RT mixture was incubated at 42 ^{o}C for 60 min. then stopped by heating at 95°C for 5 minutes. The cDNA stock was stored at -20°C.

Semi-quantitative PCR

Dream-Taq Polymerase mix (ThermoFisher-Scientific, Massachusetts, USA), was used for semi-qPCR. For each gene, the cDNA concentration and the number of PCR cycles were established in the linear amplification range. Primers were used at a concentration of 0.5 µM. PCR was performed using the DreamTaq PCR Master Mix (Life Technologies, CA, USA) on a KyraTec PCR machine (KyraTec, Australia). The PCR products were separated on 2% agarose gels in TAE buffer, stained with ethidium bromide (0.01% v/v), and photographed by the B.I.S. 202D BioImaging System (Amersham Pharmacia Biotech, USA). Intensities of the bands were quantified using the ImageJ 1.47t Image software (NIH, USA). After normalization versus the endogenous "housekeeping" genes, Crk, Tfg, Arl6ip1 and Hprt, which were the most uniformly expressed genes in all RNA samples, were subjected to global gene expression profiling (3).

Gene-specific primers were selected using the Primer blast software (NCBI, USA), and verified using Primer3 (Steve Rozen and Helen J. Skaletsky (2000) Source code available at http://fokker.wi.mit.edu/primer3/), Primers were supplied by Sigma-Aldrich (St. Louis, MO, USA). All primers for real time PCR were designed and supplied by Agentek-Israel. Primer sequences used for semi-qPCR in this study are provided below:

Gene	Sense primer	Antisense primer	Product size (bp)
Akap9	CTCAGATTAATGGCGGGAAA	TTCCGAACTCAGCTGTTCCT	170
Arl6ip1	CTCGATCCATCTGTGCTGTC	ACCGCAGCAAGAGAAATGAT	260
Bambi	CACTTTGGAATGCTGTCACG	AATGGGAACCGCTATCACAG	196
Cabyr	CACCTTGAAACGGAGATCGT	CAGCATCTGGCTTCATCAAA	183
Ccl25	GGGAATCCAGAGGACATGAA	CCTCCAGCTGGTGCTTACTC	248
Ccny	GGTTCGTTCGGACACTCTTC	TGGCATACACACTGGAAGGA	300
Cdk14	AGAGGGCACACCTTTCACAG	TTCTGCGGTTTCAGGTCTCT	270
Cdk6	CCAAATCTGCTCAACCCATC	TTCAGATGACGAGCTCATGG	251
Cep68	ACCCTCATCCCTGACAACAG	GCGAGAAAGGCATCTTAACG	185
Cldn12	GTGTGCAGATGTGCTCCTGT	CCGCAGTTTCCTCCAGTTAG	262
Crem	AGGCAAATGACCATGGAAAC	GTGTATTGCCCCGTGCTAGT	359
Crk	CGGCTCTGATTGGAGGTAAC	TTGCCATTACACTCCCCTTC	253
Cul2	TTACTCCGTGCTGTGTCCAG	ATCCCCTTCGCTGACTTCTT	320
Elavl1	AGCAATCAGCACACTGAACG	TCAAACCGGATAAAGGCAAC	237
Fzd1	CTCCAGGGTCATTTCCTTCA	ATCGGTCGGTTACTGCACTC	327
Greb1l	GCGTCTTGCAGCATATTGAA	GCCACTGCTGTTTGTTCTCA	230
Gtpbp10	GAAGGTGGAAGAGGTGGTGA	CTTGGCCGTTTTCATCAGTT	202
Insr	AATAAGTGCATCCCCGAGTG	TTCCAAGGTCTCTCCTCGAA	339
Map2k7	GCTCACCATCCTCAGAGAGC	GGTACCACTGCCCATCTCAC	230
Map3k8	TCGGATGTTCTCCTTGTTCC	GCCCACAACTCTCCAGTTTC	298
Mib1	CATCCATTGCGTGTTTCTTG	AACAGGTTGCAATGTGTCCA	264
Mtpap	TCACCTCCCATTCTTCCAAC	TGTTTTGTTCCCTTCCCTGT	229
Rock1	TGCTGCTGGATAAGTCTGGA	CCCAACCAAAGAATCTGCAT	245
Tfg	CAACTCAGGTTATGGCAGCA	GTCTGAGCTCCTGCATAGGG	229
Zeb1	TTCTGCAGCAACAAGACACC	GTGTAACTGCACAGGGAGCA	238

Real-time qPCR

Real-time PCR was carried out in triplicates either in a final volume of 20 µl, with 2 µl cDNA, 10 µl SYBR green master mix of SYBR green RT-PCR reagent kit (Life Technologies, Van Allen Way, Carlsbad, California) following calibration for linear range and primers concentration, or using TaqMan Fast Universal PCR Master Mix (Applied Biosystems, CA, USA), 2 µl of each (forward and reverse) primer with a total final concentration of 5 µM each, and 4µl DDW. PCR products were detected during PCR with the ABI PRISM 7700 Sequence Detector system (Applied Biosystems, CA, USA). Threshold cycle numbers (C_t) were determined with the Sequence Detector Software (version 1.6; Applied Biosystems, CA, USA) and transformed using the $\Delta\Delta C_t$ method as described by the manufacturer. The relative quantification values for each gene were normalized against the endogenous "housekeeping" gene Hprt.

Bioinformatic analysis of the gene expression profiling data

Genome-scale gene expression profiling data were analyzed by the GeneChip robust multiarray analysis (GCRMA) and preprocessing algorithm following median polish normalization using the Partek software (ProSoftware, Partek, St. Louis, MO). All signals below 5.0 (in log2 scale) were rounded to 5.0. Enrichment analysis (Fisher test) for gene signatures was performed using "EDanalysis" of the "R" Package for Gene Enrichment Disequilibrium Analysis:

http://cran.r-project.org/web/packages/EDanalysis/EDanalysis.pdf

Histopathology staining and analysis

FFPE liver tissue sections were stained either with Haematoxylin & Eosin (H&E) or with Haematoxylin alone. For both stainings, the deparaffinization and dehydration process steps (5 min every step) were: Xylene×2, ethanol-100%×2, ethanol-95%×1, ethanol-80%×1, ethanol-60%×1 ethanol-30%×1 and TBS×1. For H&E staining, the slides were immersed with Haematoxylin (30 seconds), rinsed in DDW and left for 5 min in tap water to develop the stain. Destaining was done by 8-12 fast dipping in acid ethanol, twice in tap water for 1 min and once in DDW for 2 min. After blotting the water from the slide, it was immersed in eosin for 30 sec. Rehydration and clearing were done inversely for deparaffinization and dehydration. The slides were air dried, a DAB mounting solution was added on the specimens and the slides were covered with a cover slip and analyzed under the Nikon Eclipse E600 light microscope equipped with an Olympus DP71 camera and Cell'A software. A similar protocol was also used for Eosin staining, DAB and Haemotoxylin steps.

Histological sections were reviewed and characterized by the pathologist (O.P.) for inflammation, mitotic incidence, tissue damage, and cell homogeneity. Liver tumors were characterized, and only those assigned as "well differentiated HCCs" were used for further experiments.

Bradford protein quantification assay

The protein content of each lysate sample was quantified by the Bradford assay. A standard curve of OD against increasing protein concentration was established for each protein sample using serial dilutions of BSA standard protein (1 mg/ml) and a Coomassie blue based dye reagent (Bio-RAD). 1 / 2.5 / 5 / 10 / 20 / 40 µl of standard lysate samples were incubated with 200 µl of Coomassie reagent (diluted 1:5 with DDW) and OD at 595nm was immediately measured by spectrophotometer.

Protein immunoblotting

Lysates were obtained from the whole liver; tissues were prepared by manual homogenization of liver tissue in liquid nitrogen and 400 µl protein lysis buffer containing 50 mM Tris pH 7.6, 150 mM NaCl, 20 mM MgCl2, 1% NP40, 1 mM DTT, 5% glycerol, 1 mM PMSF and 2.5 µl/ml of protease inhibitor cocktail. After 30 min rotation at 4°C and 20 min centrifugation at 4°C, 20,800*g, aqueous supernatant was taken to determine protein amounts by the Bradford assay. The samples were mixed with gel loading buffer (1:6), boiled, aliquoted and stored at -80°C until use. Similar protein amounts from every sample were heated (95°C, 10 min, vortex 30 sec, 3 times) with β-Mercaptoethanol and SDS-containing protein sample buffer. Samples (20 µg) were loaded onto 10-12.5% polyacrilamide gel SDS-PAGE under reducing conditions. Proteins were electrotransferred from 2-D gel to PVDF membranes, which were shaken for at least 1h at RT with a blocking solution or 1% milk and then washed in a washing solution. Incubation with primary specific antibodies: All antibodies were diluted according to the manufacturer's recommendations, and incubated o.n., 4°C except b-Actin, which was incubated for 1h at RT. After the membranes were washed (3 times, 10 min each, in a washing solution), proteins were detected by appropriate HRP-conjugated secondary antibody (1/200), followed, after washing (4 times, 10 min), by ECL detection. Photon emission was identified by photography film development. Intensities of protein bands were quantified by computerized densitometry using the Scion Image software. The membranes were stripped for 5 min with a stripping solution, washed (3 times, 5 min in a washing solution) and rehydrated with methanol for long storage.

Immunohistopathology staining.

Liver tissue paraffin-embedded sections were mounted on glass slides, deparaffinized and graduated with ethyl alcohol, as follows: Xylyne×2, ethanol-100%×2, ethanol-95%×1, ethanol-80%×1, ethanol-60%×1 ethanol-30%×1 and TBS×1 (5 min every step). Antigen retrieval was done with 0.01 M citrate buffer (pH 6.0) in a microwave (100% power for 13 min followed by 60% for 20 min) using a pressure cooker. The slides were washed 3 times with PBS or TBS and then labeled with a secondary HRP-conjugated streptavidin antibody. After washes (3 times, 10 min), color development with diaminobenzidine was done using the Zymed kit followed by Haematoxylin counterstaining immersed with Haematoxylin (1 min), rinsed in tap water and left for an additional 2 min to develop the stain . Rehydration and clearing were done inversely for deparaffinization and dehydration. The slides were covered in a similar manner as with the histopatology staining method. Below are listed the antibodies used in this study either for immunoblotting (IB) or for immunohistochemistry (IHC):

Antibody name	Manufacturer	Catalog number	Application
Beta-actin	Santa-Cruz	Sc-1616	IB
P21	BD Bioscience	556430	IHC
Anti-mouse	DAKO	K400411	IB, IHC
Anti-goat	DAKO	F025002	IB
Anti-Rabbit	DAKO	K401111	IB, IHC
γH2Ax	Millipore	05-636	IHC
TUNEL	Life Technologies	C10245	IHC
BrdU	DAKO	Mo744	IHC
F4-80	abcam	Ab6640	IHC
Total SAPK/JNK	Cell Signaling technology	#9258	IB
Phospho-SAPK/JNK	Cell Signaling technology	#9251	IB
β-catenin	BD Biosciences	610153	IHC

Assessment of Crem protein levels by immunohistochemistry and immunoblotting.

To assess Crem protein levels in hepatocytes, paraffin-embedded slides were stained for Crem (1:100) (Santa Cruz Biotechnology, SC-440, Santa Cruz, CA). Assessment of positive stained nuclei of hepatocytes cells was performed by Nicon (Nikon Instruments Inc.,1300 Walt Whitman Road, Melville, NY, U.S.A) within 100 fields per slide, magnification x200. Crem protein levels were quantified in protein lysate by Western blot using the same Crem Ab. (1:200). Beta-Actin (1:15,000) served as the housekeeping gene for normalization.

Assessment of proliferative response, DNA damage and apoptosis

To assess the proliferative response of hepatocytes, mice were injected intraperitoneally (i.p.) with BrdU (100 μgr/1 gr of mouse weight; MP Biomedicals, France), at 2 hrs before sacrifice. Formalin-fixed livers were embedded in paraffin. Antigen retrieval of FFPE liver sections was performed in citrate buffer (pH 6.0) for 20 min in a microwave; then sections were stained with the monoclonal anti-BrdU antibody (1/100) (Dako Cytomation, Tel Aviv, Israel), using a standard direct HRP method according to the manufacturer’s instructions. Similarly to the pH3 protocol (1/1000), Diaminobenzidine (DAB) Histochemistry Kits (Life Technologies) was used for color development and sections were counterstained with haematoxylin (Polysciences, Inc., 400 Valley Road Warrington, PA 18976).

Mitotic events were counted in slides stained for hematoxylin only. Assessment of a proliferative response was performed by counting positively stained hepatocytes cells within at least 20 fields per slide, magnification x200.

To assess the DNA damage in hepatocytes, FFPE slides were stained for γH2Ax (1/1000), and for the DNA damage arrest protein P21 (1/25).

To assess the apoptosis, TUNEL staining was performed using the in situ Cell Death Detection Kit (Life Technologies) as described by the manufacturer.

Cell cultures

The human HCC cell lines Huh7, Hep3b, HepaRG, and HepG2 and the murine cell lines Bnl, Bwtg, and Snu378, Snu475 (both Snu lines were generously provided by Prof. Mehmet Ozturk, Department of Molecular Biology and Genetics, Bilkent university, Ankara, Turkey) were cultured at 37^0C, 5% CO_2 in Dulbecco's modified Eagles medium (DMEM, Gibco BRL, Grand Island, NY, USA) supplemented with 10% fetal calf serum (FCS), 1% penicillin/streptomycin. The human HCC cell line Hep40 (generously provided by Prof. Mehmet Ozturk), was cultured under the same conditions, excluding supplementation by the 10% non essential amino acids
(NEAA, Sigma-Aldrich) and absence of streptomycin.

Cell proliferation assay

Cell proliferation rate was measured by proliferation assay using a xCELLigence system for real-time and label-free monitoring of cell viability (4), in duplicates. Hep40 and Huh7 cell lines were treated with 25 nM/ul of Crem siRNA, and mock or siLuc as controls. The siRNA reagent kits were purchased from Santa Cruz (sc-37700). siRNAs were transfected using the LipofectAMINE 2000 reagent (Invitrogen, Carlsbad, CA, USA) according to the manufacturer's instructions after cells were grown to 50% confluence. Briefly, 1 μL LipofectAMINE was diluted in 50 μL OptiMEMI Reduced Serum Medium (Invitrogen, Carlsbad, CA, USA), mixed gently, and incubated for 5 min at room temperature. Subsequently, a mixture of siRNA was added and incubated for 20 min. The mixture was diluted with medium and the final concentration of siRNA was 25 nM. After treatment for the indicated time period, cells were collected for further investigation. Alternatively, cells were measured for proliferation using the xCELLigence system.

Statistical analysis and calculation software.

In this study, the *in vitro* experiments were performed in triplicates, excluding the cell proliferation rate assay using a xCELLigence system, which was performed in duplicates; in the *in vivo* experiments, at

least 3 mice per group were used. All parameters were evaluated with the two-tailed t-test. Statistical evaluation of differential expression between the experimental groups was performed using the ANOVA test. A p-value of 0.05 or less was considered significant. *t*-test, correlation test, and f-test were performed using the GraphPad Prism version 6.04 for Windows (GraphPad Software, La Jolla California USA, www.graphpad.com). Stained hepatocytes IHC sections were counted by the NIS-Elements Microscope Imaging Software (Nikon Instruments Inc.1300 Walt Whitman Road, Melville, NY, U.S.A). All Western and semiQ PCR results were calculated using ImageJ (Image processing and analysis in Java , NIH, U.S.A). To evaluate the effect of PHx on tumor incidence, the Fisher exact test was calculated online (http://www.quantpsy.org/fisher/fisher.htm). The Pearson's correlation coefficient (Supp. Table 5) was calculated for all 1,256 genes with matching copy number and expression profiles. The distribution of correlation values was compared to a randomized background distribution and was found to be significantly enriched with positive correlation values (p<E-33). The randomized background distribution was generated by repeatedly (100 times) shuffling the samples in the expression dataset only and recalculating the non-sample matched correlation values. The disparity between the two distributions was estimated using the Kolmogorov-Smirnov test.

References

1. Greene AK, Puder M. Partial hepatectomy in the mouse: technique and perioperative management. J Invest Surg 2003;16:99-102.
2. Barash H, Gross ER, Edrei Y, Ella E, Israel A, Cohen I, Corchia N, et al. Accelerated carcinogenesis following liver regeneration is associated with chronic inflammation-induced double-strand DNA breaks. Proc Natl Acad Sci U S A 2010;107:2207-2212.
3. Katzenellenbogen M, Pappo O, Barash H, Klopstock N, Mizrahi L, Olam D, Jacob-Hirsch J, et al. Multiple adaptive mechanisms to chronic liver disease revealed at early stages of liver carcinogenesis in the Mdr2-knockout mice. Cancer Res 2006;66:4001-4010.
4. Ke N, Wang X, Xu X, Abassi YA. The xCELLigence system for real-time and label-free monitoring of cell viability. Methods Mol Biol 2011;740:33-43.

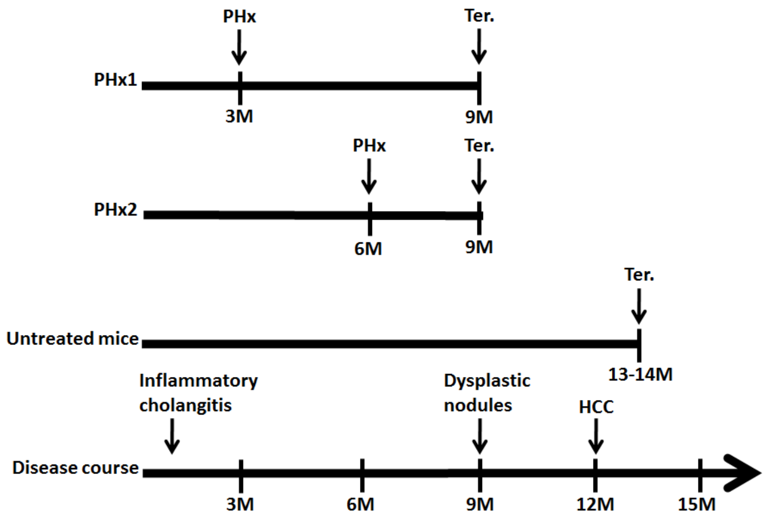

Supplementary Fig. 1: Time schedule of mouse treatments. Axis “X” below - time course of chronic liver disease for untreated Mdr2-KO/FVB males: inflammatory cholangitis & hepatitis starting from the age of one month; dysplastic nodules starting from the age of nine months; HCC starting from the age of 12 months. Untreated Mdr2-KO/FVB mice were terminated at the age of 13 months (females) or 14 months (males). 70% PHx was performed on Mdr2-KO/FVB mice of both sexes, at the age of either three or six months; then, all hepatectomized mice were terminated at the age of nine months.

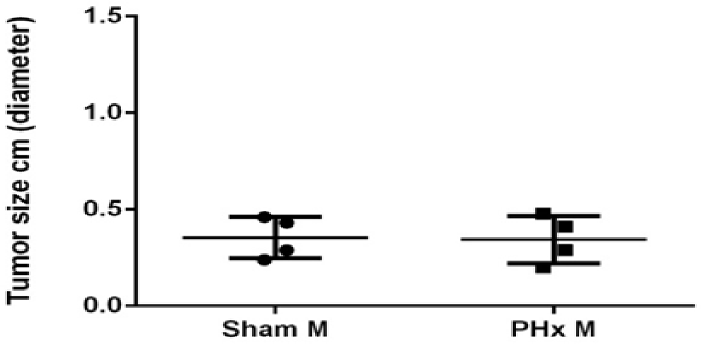

Supplementary Fig. 2: HCC development was not accelerated in the Mdr2-KO/B6 mice following 70% PHx. Tumor incidence of Mdr2-KO/B6 male mice that were subjected to 70% PHx at the age of six months and sacrificed at the age of 14 months (16 mice in the PHx group and 13 mice in the sham-operated group). Axis Y: tumor diameter, cm.

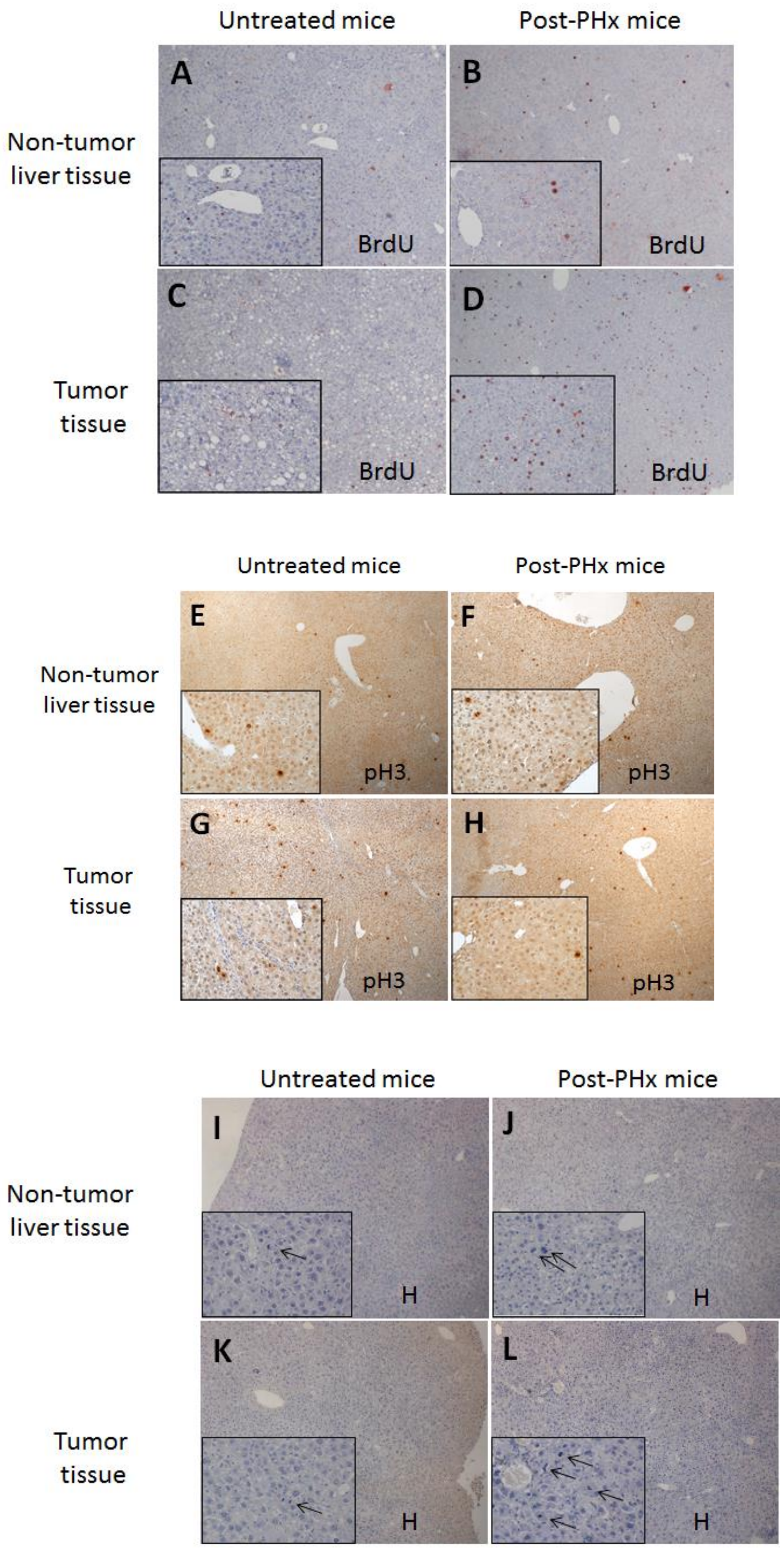
Untreated mice
Post-PHx mice
Non-tumor liver tissue
Tumor tissue
A
B
C
D
BrdU
E
F
G
H
pH3
I
J
K
L
H

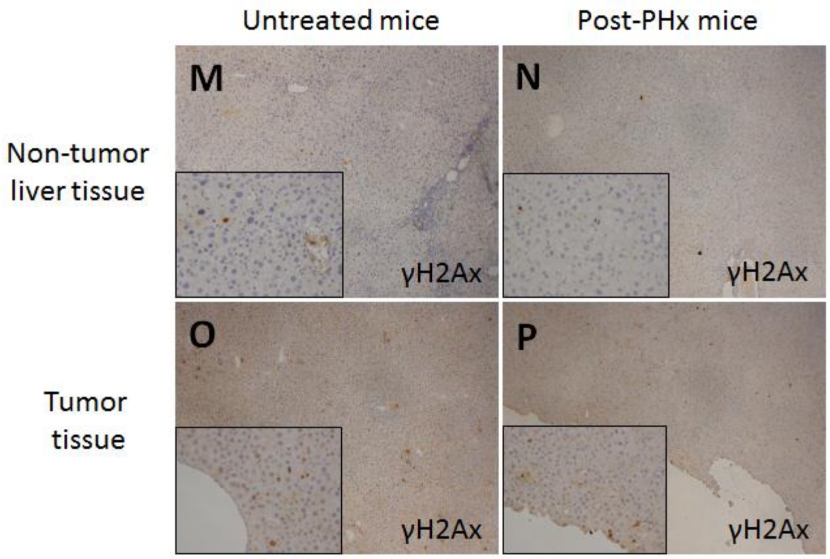

Supplementary Fig. 3: Proliferation and DNA damage markers indicate replication arrest and increased DNA repair in post-PHx livers. IHC or histological staining of tumor and their matched non-tumor liver tissues from 9-month-old post-PHx and 13-14-month-old untreated mice. (A-D) IHC staining for BrdU. (E-H) IHC staining for phospho-histone H3 (pH3). (I-L) Hematoxylin (H) staining for detection of mitotic figures. (M-P) IHC staining for phospho-histone H2A (γH2AX). Six mice per each experimental group, excluding γH2AX staining for post-PHx, where five samples were stained.

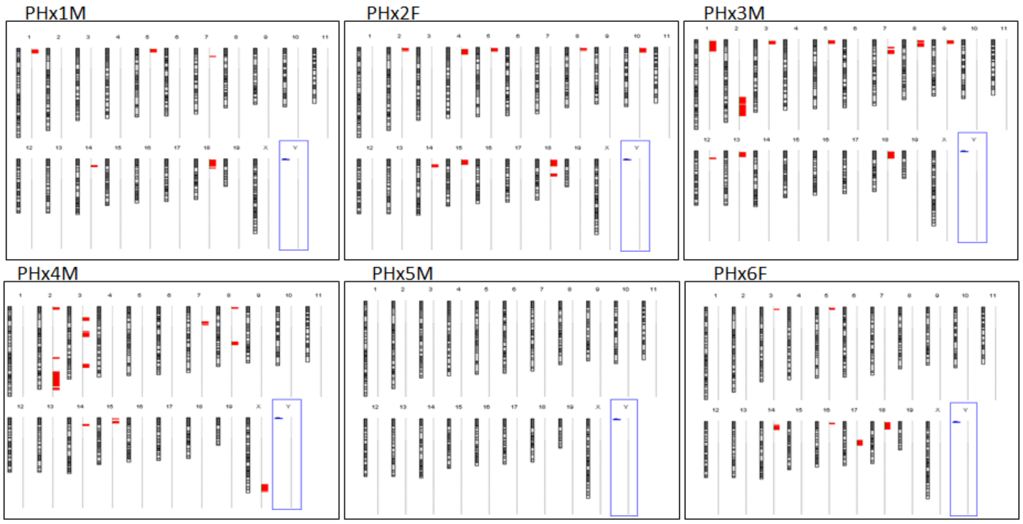

A

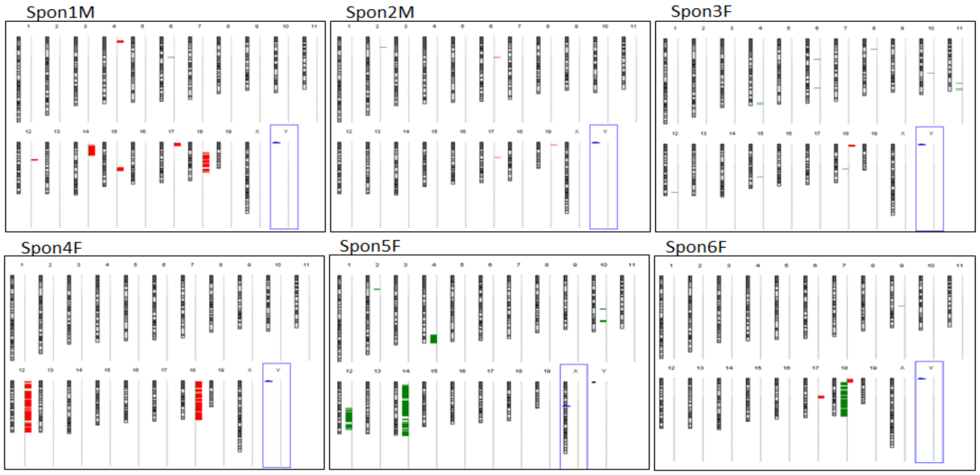

B

Supplementary Fig. 4. Distinct patterns of genomic aberrations in the post-PHx and spontaneous tumors of Mdr2-KO/FVB mice. Individual genomic aberration maps were obtained using aCGH for six post-PHx (A) and six untreated (B) mice. (A) Post-PHx tumors had only amplifications (red). (B) Spontaneous tumors from untreated mice had both amplifications (red) and deletions (green). Most amplifications in post-PHx tumors were located near centromeres (top). The aCGH maps of samples PHx1M, PHx2F, PHx3M, and PHx4M were published by us previously (Barash et al., *PNAS* 2010).

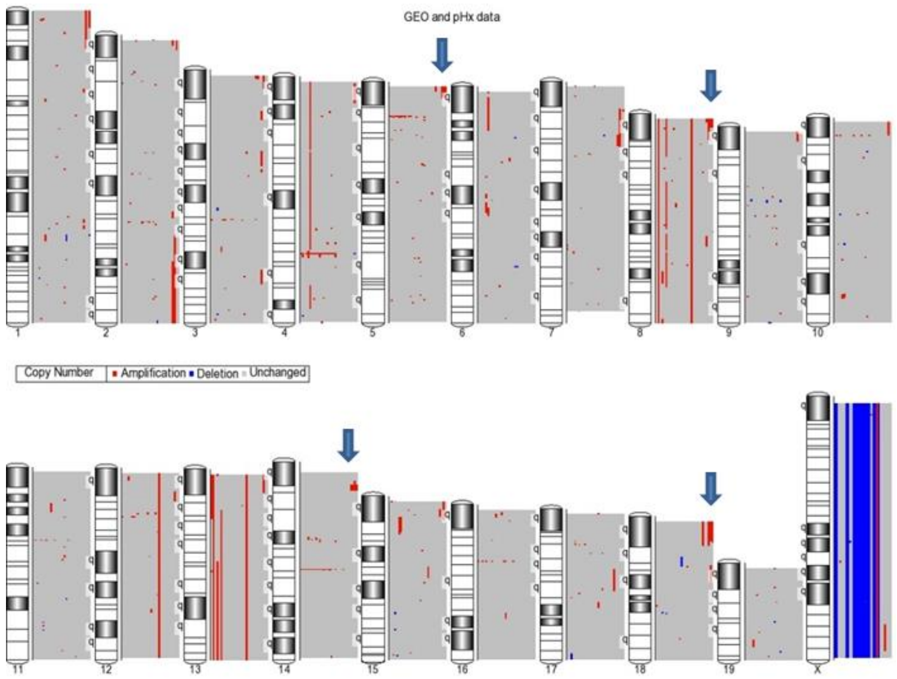

Supplementary Fig. 5: Rare overlap of chromosomal amplifications in published murine HCCs with common amplified regions in HCCs from post-PHx Mdr2-KO/FVB mice. Genomic map comparing chromosomal aberrations in the post-PHx tumors with several published HCC mouse models (GEO dadasets GPL2884, GPL4092, GPL8086). Chromosomes are indicated by chromosome number; for each chromosome, 76 mouse arrays were compared with the six post-PHx samples. Common amplified regions of post-PHx tumors are marked by arrows.

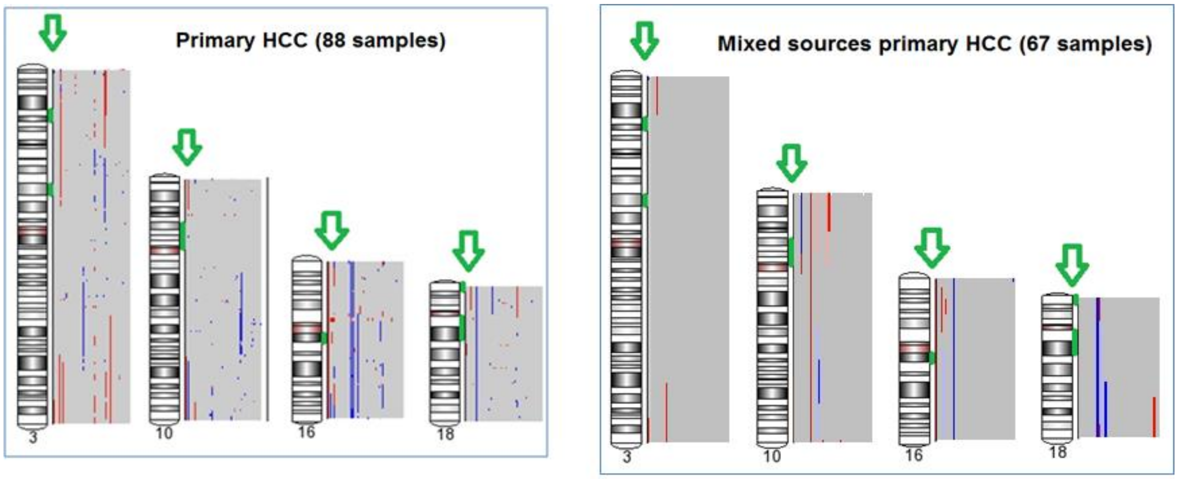

Supplementary Fig. 6: Rare overlap of chromosomal amplifications in published human HCCs with the syntenic common amplified regions in HCCs from post-PHx Mdr2-KO/FVB mice. Synteny map comparing chromosomal aberrations in murine post-PHx tumors with syntenic regions of human genome (human HCCs were from GSE8351 and GSM206985-207045 GEO datasets). Syntenic regions are marked in green; amplifications in red, deletions in blue.

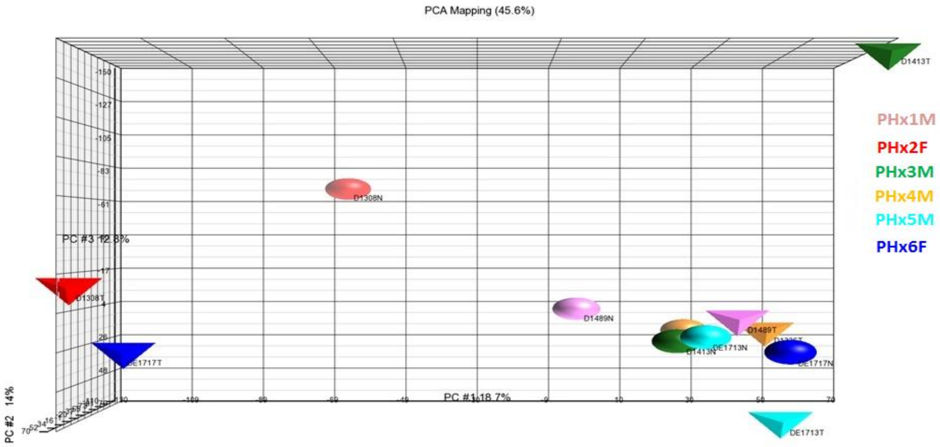

Supplementary Fig. 7: Principal Component Analysis (PCA) of gene expression data of post-PHx livers from Mdr2-KO/FVB mice. PCA mapping of the genome-scale gene expression data of six PHx-induced tumors and their matched non-tumor liver tissue samples

from Mdr2-KO/FVB mice. Each mouse is shown in a unique color; rectangles represent tumors and ovals represent non-tumor tissues.

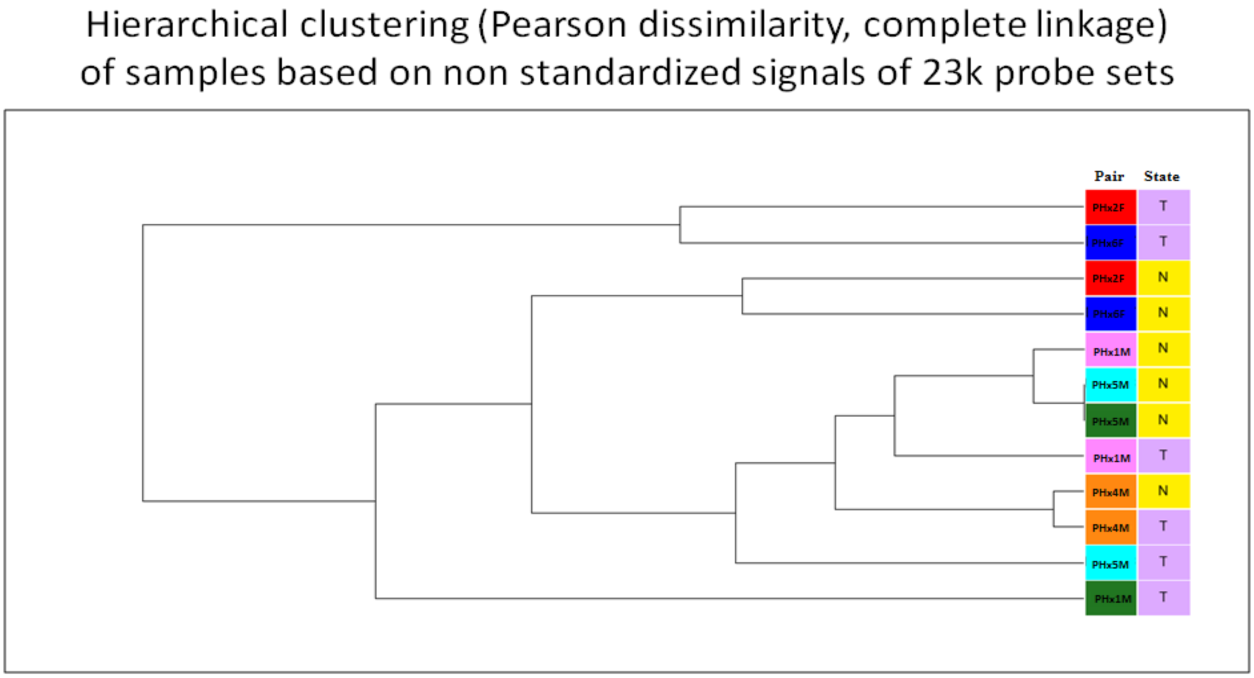

Supplementary Fig. 8: Hierarchical clustering analysis of gene expression data of the post-PHx livers from Mdr2-KO/FVB mice. Hierarchical clustering (Pearson dissimilarity, complete linkage) of samples based on non standardized signals of the 23k probe sets. Tumors from female origin (PHx2F-T & PHx6F-T) cluster together in a separate root. The non-tumor tissue of PHx4M is clustering inside the tumor roots. This non-tumor tissue is the most progressed in the tumor direction among other non-tumor tissues. Thus, the PHx4M tumor has the minimal number of aberrantly expressed genes among other PHx-induced tumors (see Supp. Table 4).

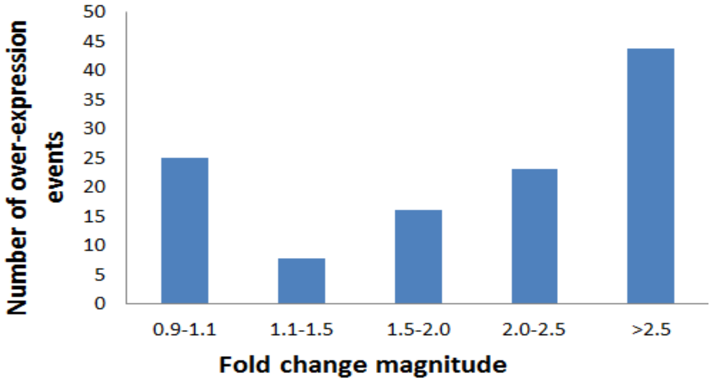

Supplementary Fig. 9: Correlation between gene amplification and expression in the post-PHx livers from Mdr2-KO/FVB mice. Relative gene expression of 22 genes, located in common amplified regions, tested by semi-qRT-PCR for 6 tumor samples (132 tests). Axis Y: number of tests where gene was over-expressed in tumor, FC threshold = 1.98. Axis X: genomic amplification (FC). Both for expression and amplification, each tumor was compared with its matched non-tumor liver tissue.

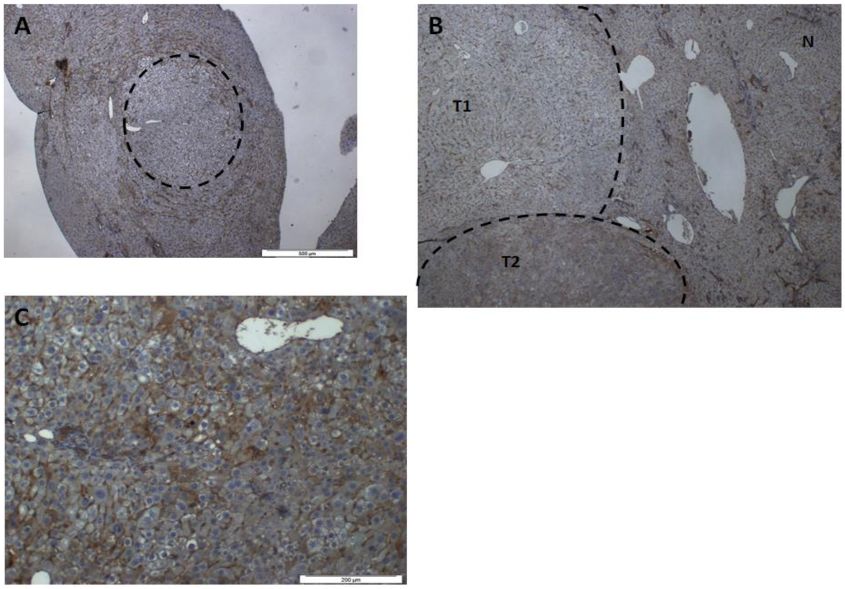

Supplementary Fig. 10: Cytoplasmic beta-catenin staining in liver nodules and tumors. IHC staining for beta-catenin in livers and tumors of the post-PHx Mdr2-KO/FVB mice. Reduced

beta-catenin levels in some nodules (dashed oval in A) and tumors (T1 in B); some tumors (T2 in B and tumor in C) and all non-tumor liver tissues were positive for cytoplasmic beta-catenin. (A) nodule; (B) tumors; (C) the only one tumor which had a gene expression signature of beta-catenin activation.

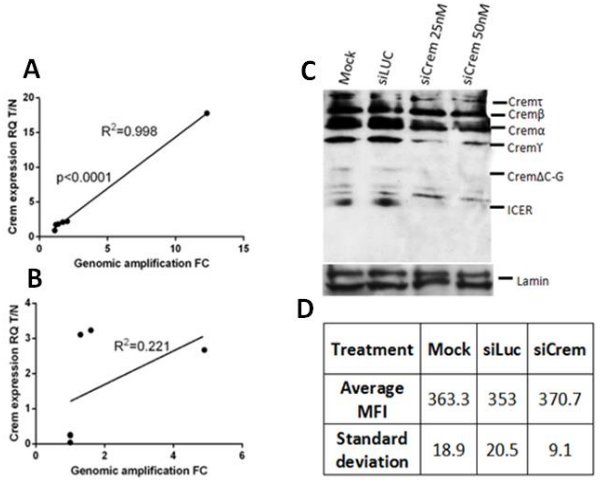

Treatment	Mock	siLuc	siCrem
Average MFI	363.3	353	370.7
Standard deviation	18.9	20.5	9.1

Supplementary Fig. 11: Crem amplification affects gene and protein expression levels, but not the hepatocyte cell size. (A-B) Axis Y: relative Crem expression tumor/non-tumor, as detected by RT-PCR. Axis X: genomic amplification of Crem (fold-change tumor/non-tumor) as detected by aCGH. Crem amplification was observed in both post PHx tumors (A), and in spontaneous tumors (B). While in both groups, Crem over-expression was observed only in livers containing genomic amplification, the correlation between gene expression and genomic amplification was significantly stronger for the post-PHx tumors. (C-D) Crem knock-down by siRNA: (C) Following siCREM transfection of the Hep40 HCC cells, most of CREM isoforms were down-regulated. CREM proteins were detected in the nuclear fraction. (D) No effect of siCREM on cell size was observed, as measured on Huh7 triplicates by FACS using a mean fluorescence intensity.

Supplemental Table 1: Increased tumor load observed in the post-PHx livers. Summary of tumor load per mouse and the relative number of mice which developed tumors when comparing to the whole group of each treatment. (A) Tumor load of mice, where only mice with tumors bigger or equal to 0.3 cm were counted for the calculation of percent of mice. For the calculation of tumor load per mouse (TL/Mouse), we counted all mice in each group. (B) Tumors larger than 0.5 cm in diameter (bold) were observed mainly for PHx-treated livers (p=0.03). For both tables, all mice were from the FVB genetic background.

A

	3mM		3mF		6mM		6mF		3mF+M		6mF+M	
	PHx	Sham	PHx	Sham	PHx	Sham	PHx	Sham	PHx	Sham	PHx	Sham
**Percent of mice with T=>0.3**	0.33	0.18	0.76	0.25	0.55	0.18	0.52	0.30	0.54	0.21	0.54	0.24
**Average TL/Mouse**	0.61	0.27	1.29	0.31	0.90	0.18	0.68	0.30	0.94	0.29	0.78	0.24
**STDV**	1.09	0.63	0.92	0.60	1.02	0.4	0.80	0.48	1.06	0.61	0.90	0.44
**TTEST**	**0.228**		**0.001**		**0.034**		**0.173**		**0.002**		**0.012**	

B

Operation age	Gender	Treatment	Number of Mice	Tumor load per mouse for tumors of different size ranges (tumor diameter, cm)					
				>1.2	1.2-1.0	1.0-0.8	0.7-0.5	0.5-0.4	<=0.3
3 months	Females	PHx	17	0	0	**0.06**	**0.29**	0.41	3.53
		Sham	16	0	0	0	0	0.19	2.88
	Males	PHx	18	0	**0.06**	**0.06**	**0.17**	0.17	2.17
		Sham	22	0	0	0	0	0.05	1.09
6 months	Females	PHx	25	**0.04**	0	**0.08**	**0.08**	0.28	3.68
		Sham	10	0	0	0	**0.1**	0.1	1.9
	Males	PHx	20	0	0	**0.1**	**0.05**	0.25	3.75
		Sham	11	0	0	0	0	0.09	1.27

Supplemental Table 2: Common amplifications in spontaneous (Spon) HCC tumors of the untreated Mdr2-KO/FVB mice. Status in the spontaneous tumors of the chromosomal regions that were frequently amplified in the PHx-induced tumors of the Mdr2-KO/FVB mice. The common amplification in chromosome 18 was the most prevalent.

**Chromosome**	**Spon1M**	**Spon2M**	**Spon3F**	**Spon4F**	**Spon5F**	**Spon6F**
**14**	**Amp.**				**Del.**	
**18**			**Amp.**	**Amp.**		**Amp.**

Supplemental Table 3: Amplifications in human HCC. Human HCC studies where amplifications of chromosomal regions orthologous to frequently amplified regions in post-PHx HCC tumors of Mdr2-KO mice have been found.

Murine	Human	(1)	(2)	(3)	(4)	(5)	(6)	(7)	(8)	(9)	(10)
14qA1-2	3p							V		V	
14qA1-2	3p14.1		V								
14qA1-2	3p24.1		V								
14qA1-2	3p24.2		V								
5qA1	7q				V		V	V	V	V	
5qA1	7q21.13		V								
5qA1	7q21.2		V								
18qA1	10p							V		V	
18qA1	10p11.21		V	V							V
18qA1	10p11.22		V	V							V
18qA1	10p11.23		V	V							V
18qA1	10p12.1		V	V							
18qA1	18p11.32		V								V
18qA1	18q									V	
18qA1	18q11.1		V								

18qA1	18q11.2		V			V					
8qA1.1	19p	V			V				V		
8qA1.1	19p13.2		V								

Supplemental Table 4: Numbers of differentially expressed genes in the post-PHx tumors of Mdr2-KO/FVB mice. Summary of total differentially expressed genes (DE), including up-regulated and down-regulated genes for each tested PHx-induced tumor. The threshold of minimal 1.8- fold change (tumor/non-tumor) was used. The last letter in each tumor ID indicates mouse gender: M – males, F - females.

Tumor ID	PHx1M	PHx2F	PHx3M	PHx4M	PHx5M	PHx6F
Mouse ID	D1489	D1308	D1413	D1336	DE1713	DE1717
Total DE genes	338	1314	829	126	261	1215
Up-regulated	155	680	387	96	113	458
Down-regulated	183	634	442	30	148	757

Supplemental Table 5: Observed number of over-expressed genes in the amplified regions of PHx-induced HCC tumors was significantly higher than randomly expected. The Pearson correlation between gene amplification and gene expression was calculated for 1,256 genes which where amplified in at least one post-PHx tumor (n=6). For each correlation range, the number of over-expressed genes in amplified regions, as detected by expression microarray, is represented in column “Observed”. Alternatively, Pearson's correlation was calculated by randomization of the expression microarray data, followed by comparison to the 1,256 amplified genes (column “Calculated”).

A significant enrichment in the number of co-amplified and overexpressed genes was detected for observed data, when comparing to the expected number calculated using data randomization of gene expression ($p<0.001$).

Pearson's correlation range	Observed	Calculated
0.7 - 0.8	50	16
0.8 - 0.9	47	8
0.9 - 1.0	44	14

Supplemental Table 6: Confirmation of the direct correlation of gene amplification with expression by semi-qRT-PCR. Semi-quantitative RT-PCR was performed to measure the fold change in expression (Exp. FC) of 22 genes selected out of the 32 candidate genes located in the common amplified regions. For each sample, the amplification fold change (Amp. FC) is also shown. Expression FC was calculated relatively to the average expression of the three housekeeping genes (bottom rows).

Gene symbol	PHx1M		PHx2F		PHx3M		PHx4M		PHx5M		PHx6M	
	Exp. FC	Amp. FC	Exp. FC	Amp. FC	Exp. FC	Amp. FC	Exp. FC	Amp. FC	Exp. FC	Amp. FC	Exp. FC	Amp. FC
Akap9	1.1	1.5	5.4	2.0	1.4	3.3	2.1	1.0	0.9	1.0	1.2	1.6
Bambi	1.2	1.4	2.8	12.3	8.9	1.7	1.9	1.2	0.3	1.1	0.6	2.0
Cabyr	0.9	1.4	32.4	3.0	1.3	1.7	2.7	0.9	0.2	1.1	2.1	2.0
Ccl25	0.9	1.4	0.9	1.3	0.7	2.1	1.3	1.6	1.1	1.0	1.3	1.1
Ccny	1.7	1.4	3.3	3.1	1.0	1.7	1.6	0.9	0.7	1.1	1.6	2.0
Cdk14	0.6	1.5	0.5	2.0	0.9	3.3	1.3	1.0	0.3	1.0	0.6	1.6
Cdk6	1.4	1.5	1.1	2.0	0.8	3.3	2.4	1.0	1.2	1.0	1.2	1.6
Cep68	0.6	1.0	1.5	1.0	1.5	1.0	2.4	1.0	0.6	1.0	0.7	1.0
Cldn12	1.3	1.5	1.3	2.0	0.9	3.3	1.4	1.0	1.2	1.0	1.0	1.6
Crem	1.3	1.4	3.6	12.3	2.5	1.7	1.6	1.2	0.4	1.1	2.1	2.0
Cul2	1.2	1.4	1.3	12.3	1.1	1.7	1.1	1.2	0.9	1.1	1.2	2.0
Elavl1	0.6	1.4	1.7	1.3	1.2	2.1	1.2	1.6	1.3	1.0	1.1	1.1
Fzd1	1.6	1.5	0.8	2.0	1.0	3.3	2.0	1.0	1.0	1.0	0.9	1.6
Greb1l	0.5	1.4	1.5	3.1	1.6	1.7	2.5	0.9	0.6	1.1	0.7	2.0
Gtpbp10	1.1	1.5	3.1	2.0	1.0	3.3	1.9	1.0	0.8	1.0	1.0	1.6
Insr	1.1	1.4	1.4	1.3	1.2	2.1	1.2	1.6	1.1	1.0	0.9	1.1
Map2k7	2.3	1.4	1.5	1.3	2.0	2.1	1.9	1.6	1.8	1.0	1.2	1.1
Map3k8	0.9	1.4	0.6	12.3	1.0	1.7	1.2	1.2	0.8	1.1	1.0	2.0
Mib1	1.8	1.4	2.3	3.1	1.0	1.7	2.0	0.9	0.7	1.1	1.2	2.0
Mtpap	0.8	1.4	11.4	3.1	0.8	1.7	2.4	1.2	0.2	1.1	10.5	2.0
Rock1	0.8	1.4	1.9	3.1	1.3	1.7	1.3	0.9	1.3	1.1	1.1	2.0
Zeb1	1.2	1.4	36.4	3.1	0.9	1.7	2.0	1.2	0.3	1.1	1.7	2.0
Housekeeping genes												
Arl6ip1	1.2	1.0	1.0	1.0	1.0	1.0	1.1	1.0	0.8	1.0	1.1	1.0
Crk	1.1	1.0	1.1	1.0	1.1	1.0	1.0	1.0	1.0	1.0	1.0	1.0
Tfg	1.2	1.0	1.1	1.0	1.5	1.0	0.8	1.0	1.0	1.0	1.1	1.0

Supplemental Table 7: Analysis of signaling pathways by GO. Enhanced Wnt signaling pathway was observed in the common amplified regions. GO analysis was applied for 122 genes observed in the common amplified regions of the post-PHx tumors. The number of differentially expressed genes in the common amplified regions that were involved in each pathway is indicated (there may be an overlap between genes from different pathways).

Pathway	Number of genes
WNT Signaling	12
Rho GTPases	5
MAPK signaling	4
G12-G13 in Cellular Signaling	5
JAK-STAT pathway	4
JNK	3
PKA	3
GSK3 Signaling	3
Nanog in Mammalian ESC Pluripotency	3
NF-kB	3

Supplemental Table 8: The Wnt signaling signature in PHx-induced tumors. The Wnt signaling activation signature was observed in the only one tumor, PHx5M, containing no genomic amplifications. Relative tumor/non-tumor gene expression was measured using microarray data: ▼ is for down regulated and ▲ is for upregulated expression in tumors.

Gene	PHx1M	PHx2F	PHx3M	PHx4M	PHx5M	PHx6F
Axin2					▲	
Lect2	▼		▼		▲	
Rhbg					▲	
Glul					▲minor	
Lgr5					▲	
Rnase4					▲minor	
Igfbp1				▲	▲	
Tbx3			▼	▼	▲	▼

Supplemental Table 9: The CREM-specific siRNAs used in this study target most of the known CREM protein isoforms. Known Crem protein isoforms and their targeting by the individual siRNAs. SiRNAs Si1,2,3 were used all together as a mixture for the CREM knockdown assay in HCC cell lines.

Dalton	aa	Isoform [UniParc].	Si3	Si2	Si1
**38,940**	361	Isoform 1 (CREM-BCEFGgammaHIbeta)	v	v	v
**37,628**	349	Isoform 2 (Beta) (CREM-BCEFGHIbeta)			
**37,577**	348	Isoform 3 (CREM-BCEFGIalpha) (Tau)			
**26,320**	237	Isoform 4 (Alpha) (CREM-BEFHIb) (CREM-gamma)	v	v	v
**26,513**	245	Isoform 5 (CREM-theta2tau-gamma) (CREM-theta2EFGHIb)	v	v	v
**13,435**	120	Isoform 6 (CREM2alpha-a) (IcergammaHIalpha) (ICER1) (ICERI)	v	v	v
**12,124**	108	Isoform 7 (CREM2alpha-b) (IcerHIalpha) (ICERIgamma)	v	v	v
**13,486**	121	Isoform 8 (CREM 2beta-a) (IcergammaHIbeta) (ICERII)	v	v	v
**12,175**	109	Isoform 9 (CREM 2beta-b) (IcerHIbeta) (ICERIIgamma)	v	v	v
**29,656**	270	Isoform 10 (CREMtheta1tau2beta) (CREM-theta1EFGHIbeta)	v	v	v
**24,774**	219	Isoform 11 (CREM-theta1beta) (CREM-theta1EFHIbeta)	v	v	v
**21,630**	194	Isoform 12 (CREM-theta2beta) (CREM-theta2EFHIb) (CREM-theta2-gamma)	v	v	v
**33,825**	312	Isoform 13 (CREM-BEFGgammaHIbeta)	v	v	v
**32,514**	300	Isoform 14 (CREM-tau2-gamma) (CREM-BEFGHIbeta)	v	v	v
**23,839**	221	Isoform 15 (CREM-BGHIbeta)	v	v	v
**18,927**	170	Isoform 16 (CREM-deltaC-G) (CREM-BgammaHIbeta)	v	v	v
**31,405**	286	Isoform 17 (CREM-BHIbeta)	v	v	v
**31,354**	285	Isoform 18 (CREM-BHIalpha)	v	v	v
**30,968**	282	Isoform 19 (CREM-theta1tau2gamma) (CREM-theta1EFGgammaHIbeta)	v	v	v
**11,535**	102	Isoform 20	v	v	v
**12,679**	112	Isoform 21	v	v	v
**14,871**	137	Isoform 22	v	v	v
**27,549**	248	Isoform 23	v	v	v

References

1. Crawley JJ, Furge KA. Identification of frequent cytogenetic aberrations in hepatocellular carcinoma using gene-expression microarray data. Genome Biol 2002;3:RESEARCH0075.
2. Guan XY, Fang Y, Sham JS, Kwong DL, Zhang Y, Liang Q, Li H, et al. Recurrent chromosome alterations in hepatocellular carcinoma detected by comparative genomic hybridization. Genes Chromosomes Cancer 2000;29:110-116.
3. Hashimoto K, Mori N, Tamesa T, Okada T, Kawauchi S, Oga A, Furuya T, et al. Analysis of DNA copy number aberrations in hepatitis C virus-associated hepatocellular carcinomas by conventional CGH and array CGH. Mod Pathol 2004;17:617-622.
4. Kakar S, Chen X, Ho C, Burgart LJ, Sahai V, Dachrut S, Yabes A, et al. Chromosomal changes in fibrolamellar hepatocellular carcinoma detected by array comparative genomic hybridization. Mod Pathol 2009;22:134-141.
5. Katoh H, Shibata T, Kokubu A, Ojima H, Loukopoulos P, Kanai Y, Kosuge T, et al. Genetic profile of hepatocellular carcinoma revealed by array-based comparative genomic hybridization: identification of genetic indicators to predict patient outcome. J Hepatol 2005;43:863-874.
6. Laurent-Puig P, Zucman-Rossi J. Genetics of hepatocellular tumors. Oncogene 2006;25:3778-3786.
7. Midorikawa Y, Yamamoto S, Tsuji S, Kamimura N, Ishikawa S, Igarashi H, Makuuchi M, et al. Allelic imbalances and homozygous deletion on 8p23.2 for stepwise progression of hepatocarcinogenesis. Hepatology 2009;49:513-522.

8. Poon TC, Wong N, Lai PB, Rattray M, Johnson PJ, Sung JJ. A tumor progression model for hepatocellular carcinoma: bioinformatic analysis of genomic data. Gastroenterology 2006;131:1262-1270.

9. Raidl M, Pirker C, Schulte-Hermann R, Aubele M, Kandioler-Eckersberger D, Wrba F, Micksche M, et al. Multiple chromosomal abnormalities in human liver (pre)neoplasia. J Hepatol 2004;40:660-668.

10. Schlaeger C, Longerich T, Schiller C, Bewerunge P, Mehrabi A, Toedt G, Kleeff J, et al. Etiology-dependent molecular mechanisms in human hepatocarcinogenesis. Hepatology 2008;47:511-520.
